# Association Between Vitamin D Exposure and Head and Neck Cancer: A Systematic Review With Meta-Analysis

**DOI:** 10.3389/fimmu.2021.627226

**Published:** 2021-02-23

**Authors:** Yuting Pu, Gangcai Zhu, Yimin Xu, Siyuan Zheng, Bin Tang, Huimei Huang, Irene X. Y. Wu, Donghai Huang, Yong Liu, Xin Zhang

**Affiliations:** ^1^ Department of Otolaryngology-Head and Neck Surgery, Xiangya Hospital, Central South University, Changsha, China; ^2^ Clinical Research Center for Pharyngolaryngeal Diseases and Voice Disorders in Hunan Province, Changsha, China; ^3^ Otolaryngology Major Disease Research Key Laboratory of Hunan Province, Changsha, China; ^4^ Department of Otolaryngology-Head and Neck Surgery, The Second Xiangya Hospital, Central South University, Changsha, China; ^5^ Department of Epidemiology and Health Statistics, Xiangya School of Public Health, Central South University, Changsha, China; ^6^ National Clinical Research Center for Geriatric Disorders (XiangYa Hospital), Changsha, China

**Keywords:** vitamin D, head and neck cancer, meta-analysis, observational studies, prevention, prognosis

## Abstract

**Background:**

Vitamin D deficiency is a well-described preventable cause of many cancers; the association of vitamin D use with the development of head and neck cancer (HNC) is not clear. We aim to conduct a systematic review of the studies assessing the relation between vitamin D exposure and the prevention and prognosis of the HNC using meta-analysis.

**Methods:**

PubMed, EMBASE, Cochrane Library, Web of Science up to 1 January 2021, and reference lists of related studies were searched. We extracted observational studies reporting the association between vitamin D (vitamin D receptor gene polymorphisms, 25-hydroxyvitamin D concentrations, and vitamin D intake) and the outcomes of interest (HNC incidence and HNC mortality) in HNC patients aged 18 or older. Fixed effects models were used to calculate pooled effect sizes and 95% confidence intervals (CIs) by RevMan (version 5.3).

**Results:**

Sixteen studies with a total of 81,908 participants were enrolled in our meta-analysis. Based on the pooled genomic analysis, comparing with participants with the genotypes of *Ff* + *FF* or *FF*, the pooled odds ratio (OR) of participants with the genotype of *ff* was 0.77 (95% CI: 0.61 to 0.97) and 0.75 (0.58 to 0.97), respectively. A similar trend was noted when comparing *tt* with *Tt* + *TT* or *TT*, in which OR (95% CI) was 0.70 (0.55 to 0.90) and 0.72 (0.55 to 0.95). No significant association was identified between *BsmI* polymorphism and HNC. Furthermore, the OR of HNC incidence was 0.77 (0.65 to 0.92) for participants with vitamin D intake over the ones with a regular diet. High concentrations of circulated 25-hydroxyvitamin D (25-OHD) significantly decreased by 32% of HNC incidence (OR (95% CI): 0.68 (0.59 to 0.78)) and increased HNC survival (pooled hazard ratio 1.13, 1.05 to 1.22) during a 4–5 years follow-up. High concentrations of circulating 25-OHD in patients with HNC led to a decreased risk of mortality to 0.75 (0.60 to 0.94) as the follow-up extends to 8–12 years.

**Conclusions:**

Elevated activities of vitamin D by diet intake, genomic polymorphisms, or circulated 25-OHD may protect people from HNC and improve the prognosis of patients with HNC.

**Systematic Review Registration:**

PROSPERO, identifier CRD42020176002 (https://www.crd.york.ac.uk/PROSPERO/display_record.php?RecordID=176002).

## Introduction

Head and neck cancer (HNC) is the sixth most common cancer, with approximately >700,000 new cancer cases and 350,000 deaths worldwide annually; more than 90% of HNC are squamous epithelial cell carcinomas arising from the oral cavity, larynx, oropharynx, and hypopharynx (1–4). Cigarette smoking, alcohol drinking, and infection with high-risk types of human papillomavirus (HPV) are major risk factors for patients with HNC (5, 6). In recent years, treatments such as surgical resection, radiotherapy, chemotherapy, and immunotherapy have been improved and matured, conferring patients a certain degree of survival benefit. However, the prognosis for patients with HNC is still poor; 5-year overall survival is only 40 to 50% (7–11). Cancer prevention is a fundamental and effective means to reduce cancer incidence and mortality. Interestingly, the antitumor effects of immunonutrition led to improve quality of life and better survival in HNC patients (12). Supplemental vitamins, including vitamin D, have been advocated for the prevention of various cancers in recent years (13, 14). Therefore, it would be of considerable significance to investigate the potential associations between vitamin D and HNC.

Skin synthesis after sun exposure and dietary intake are the primary natural sources of vitamin D in humans. The ingested or synthesized vitamin D will be correspondingly hydroxylated in the liver and kidney to form 25-hydroxyvitamin D (25-OHD) and 1,25(OH)_2_D; 1,25(OH)_2_D is the main active form of vitamin D in circulation; its classic functions involves calcium transport and regulation of genomic transcriptions. 1,25(OH)_2_D binds to the vitamin D receptor (*VDR*) and then modulates the expression of numerous target genes, therefore, participates in the regulation of diverse cell behaviors including cell differentiation, proliferation, angiogenesis, metastasis, and immunity (15). The single nucleotide polymorphism (SNP) of the *VDR* gene affects the function of vitamin D, further confirming the role of vitamin D in cancer progression (16, 17). It has been reported that polymorphism *FokI* (rs2228570), located in the transcriptional initiation site of *VDR* gene, leads to the formation of a protein isoform and plays a vital role in the post-transcriptional modification (18). *BsmI* (rs1544410) and *TaqI* (rs731236) SNPs, located near the 3′ end of the *VDR* gene, do not change the amino acid sequence of the encoded protein but determines protein level *via* regulating the stability of *VDR* mRNA (19).

The first study focusing on the associations between vitamin D and cancer risk assumes that geographical diversity in cancer incidence may be attributed to the differences in sunlight exposure and vitamin D status (20). Since then, accumulated observational studies have further confirmed the inverse correlations of vitamin D with distinct cancer types such as colorectal, breast, and prostate cancer (21–23).

Nevertheless, it is uncertain whether vitamin D is associated with the incidence and mortality of patients with HNC, due to the inconsistent findings in previous individual investigations [some investigators propose vitamin D deficiency may raise the risk and morality of HNC (24–28); while others do not confirm the significant beneficial actions of vitamin D with compelling data (29–35)]. A recent systematic review has qualitatively summed the evidence for the effect of 25(OH)D levels on HNC etiology and outcome (36). Here, we aimed to perform a systematic review and meta-analysis of observational studies to investigate the association of vitamin D exposure in three dimensions (diet intake, circulated level, and genomic phenotype) with the incidence and mortality of HNC in the HNC patients. Our results supported the notion that elevated activities of vitamin D from diet intake, genomic polymorphisms, or high concentrations of circulated 25-OHD may protect candidates from HNC and improve the prognosis of patients with HNC.

## Methods

### Protocol and Guidance

The protocol of this meta-analysis has been registered (CRD42020176002) with the International Prospective Register of Systematic Reviews (PROSPERO). We followed the Preferred Reporting Items for Systematic Reviews and Meta-Analyses (PRISMA) guidelines to design, analyze, and report our meta-analytic findings (37). We also followed the PRISMA 2020 updated guidance. Additionally, the grading quality of this meta-analysis was reported and evaluated by using the GRADE (grading of recommendations assessment, Development and evaluation) approach (38).

### Inclusion Criteria

Available studies including case–control, retrospective, and prospective cohorts were enrolled in our analysis once the following inclusion criteria were satisfied: 1) enrolled a clinical and histological diagnosis of adults (aged 18 or older) with HNC (including corresponding control groups); 2) information regarding vitamin D exposures was provided, such as dietary intake, additional supplements of vitamin D, 25-OHD, and *VDR* gene polymorphisms; 3) incidence, mortality, or survival data for patients with HNC were clear-defined; 4) the odds ratio (OR), relative risk (RR), and hazard ratio (HR) estimate with 95% confidence intervals (CIs) (or data to calculate these) of interest outcomes were also reported.

### Exclusion Criteria

We excluded studies based on the following rules: 1) Ecologic studies, case reports, case series, reviews, editorials, letters, conference papers, and articles available in an abstract form (where the authors could not be contacted); 2) Published in non-English; 3) Studies with insufficient information for data extraction.

### Search Strategy

We conducted a systematic review of articles published before March 2020 from four databases, including PubMed, EMBASE, Web of Science, and Cochrane library. Moreover, ClinicalTrials.gov and the World Health Organization International Clinical Trials Registry Platform were searched to identify the ongoing or unpublished eligible trials. Medical Subject Headings (MeSH) including “Head and Neck Neoplasms” and “Vitamin D” was used to choose qualified studies, which was also combined with the keyword in headers and abstracts. Besides, manual searches of references cited in all selected studies and published reviews were also performed to identify additional relevant studies comprehensively. To assess the associations between vitamin D exposures and the outcomes of interest, we did not limit the publication date in our initial search strategy. We updated searches to 1 January 2021. Details of our search strategy were provided in **Supplementary Table 1**.

### Study Selection and Data Extraction

Two investigators independently performed the initial screening of potentially eligible records. After the removal of the duplicates, studies with irrelevant titles or abstracts were also excluded. Consequently, full-text screening was performed in the remained eligible reports. All disagreements were resolved by consensus. Baseline characteristics and outcomes were extracted independently from the selected articles by two investigators and cross-checked to reach an agreement. The following information was recorded in detail: the last name of the first author, publication year, the country where the study was performed, study design, sex, age at baseline, study period, measure and range of exposure, sample size (cases and controls or cohort size), description of essential baseline confounders, primary cancer location, and outcomes of interest. Articles were categorized based on the results of interest: primary outcome (HNC incidence), secondary outcome (HNC mortality and HNC survival). Articles reporting on multiple developments were included. If necessary, the primary authors were contacted to retrieve additional information.

### Study Quality

The quality of each study was independently assessed by two investigators using the Newcastle–Ottawa Scale, in which a star system was applied (with a maximum of nine stars) to evaluate a study in three domains: the selection of participants, comparability of study groups, and exposure. Finally, studies with a score of nine stars were at low risk of bias, studies with seven or eight stars were at medium risk, and those that scored six or less were at high risk of bias.

### Data Synthesis

We assessed the strength of associations between the *VDR* gene polymorphisms (*FokI*, *BsmI*, and *TaqI*), concentrations of 25-OHD, vitamin D intake and HNC. Effect sizes (OR, RR, and HR) and 95% CIs were calculated to evaluate the associations between vitamin D exposures and HNC events. If available, multivariate models were given a priority for the accurate estimate for the effects of vitamin D. Comparison of the bottom versus the top of the baseline distribution of vitamin D exposure levels was selected in each study (the lowest exposure level as reference). If the highest exposure category was used as a reference in the original research, the effect size would be inverted or recalculated. If HRs and CIs were not available, Engauge Digitizer 10.9 was used to derive estimates from survival curves. The standard error of the natural logarithm (ln) of the effect sizes was calculated from the 95% CIs using the following formula: ln [upper limit of CI] − ln [lower limit of CI])/3.92. We assumed HR and OR to approximate the same measure of RR. When the incidence was low, HR, OR, and RR were like each other. If the effect sizes of subgroups were reported separately, different subgroups could be regarded as independent studies. If 25-OHD levels were quantified in ng/ml or vitamin D intake in ng/d, the values were uniformly converted into nmol/L or IU/d. The association between the three *VDR* SNPs and HNC risk was assessed under five genetic models: the allele model, the homozygous model, the heterozygous model, the recessive model, and the dominant model.

All meta-analyses were done with Review Manager 5.3. Forest plots were used to assess and visualize the pooled estimates and corresponding 95% CIs. A *P <*0.05 was considered statistically significant. The Hardy–Weinberg equilibrium (HWE) in controls was tested using the goodness-of-fit χ^2^ statistic with one degree of freedom. Statistical heterogeneity among studies was evaluated with Q and *I^2^* statistics. If Q-test reported a *P*-value <0.1 or *I^2^* >50%, it would be defined as significant heterogeneity, which means the random effects model would be applied to pool the results. Otherwise, the fixed effects model would be applied. Subgroup analyses were performed based on geographic region, quality, cancer subsites, participant numbers, and study design of included studies to avoid the potential bias influence. Sensitivity analyses were performed by excluding each study or the studies with low quality to evaluate the stability of results. Where possible, we evaluated publication bias by plotting a funnel plot; publication bias was determined by the funnel plot with an asymmetrical shape (39, 40).

## Results

**Figure 1:**
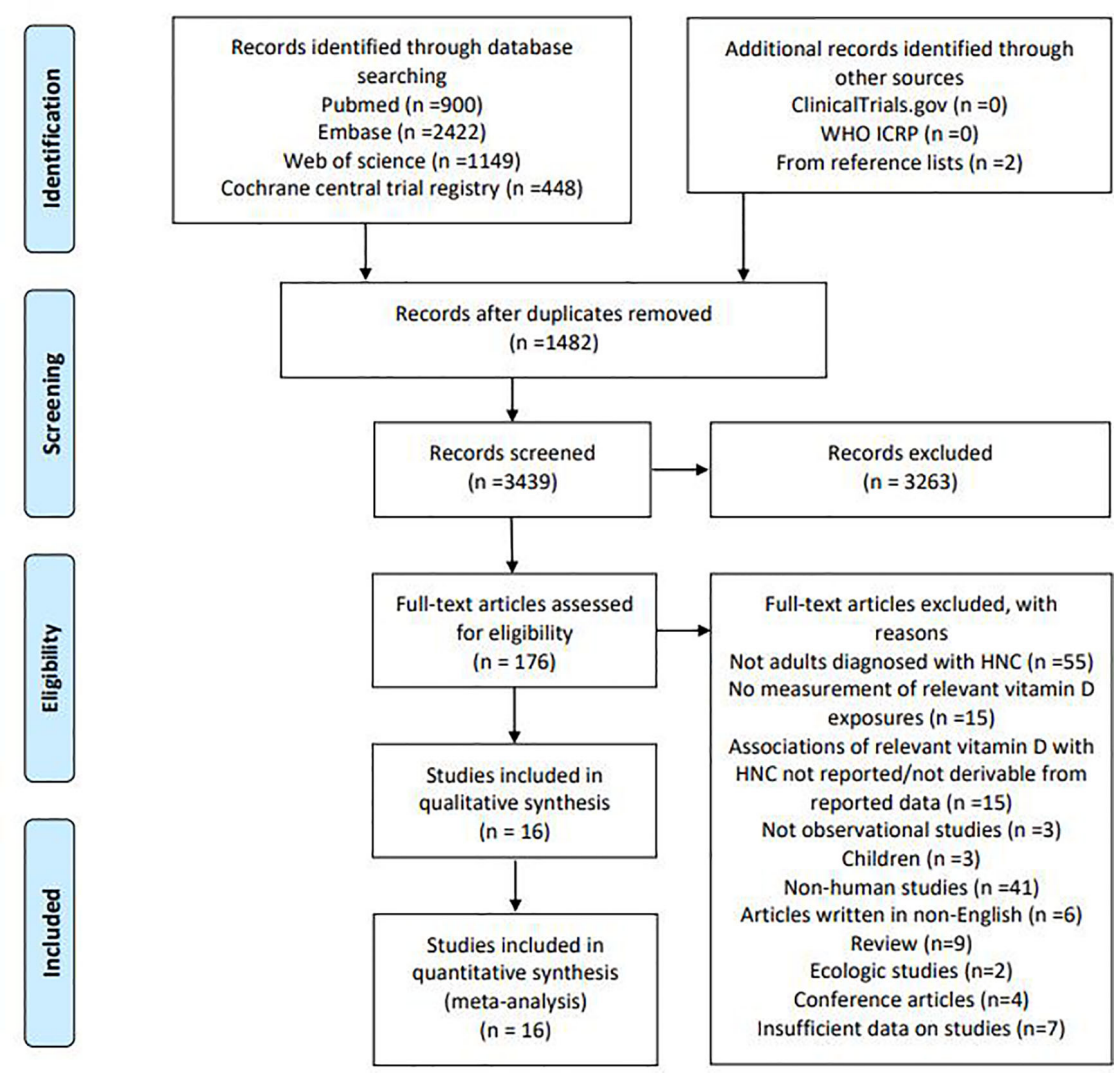
Search strategy and final included and excluded studies.

Total searches yielded 4,921 entries. After the removal of 1,482 duplicates, 3,439 titles and abstracts were assessed; 176 articles appeared to be potentially enrolled in the review. Following a full-text review, 160 articles were excluded, leaving 16 articles for final analyses, including four studies on *VDR* gene polymorphisms, nine on blood 25-OHD levels, and three on vitamin D intake. A flow diagram of our literature search strategy was shown in **Figure 1**. List of excluded articles was provided in **Supplementary Table 2**.

### Study Characteristics

Sixteen observational studies were included in our meta-analysis (24–35, 41–44), including nine case–control and seven cohort studies that were published between 2000 and 2018. Of these 16 studies, 10 were conducted in Europe, three in North America, and three in Asia-Pacific. In the 81,908 participants, 2,270 participants possessed the information of *VDR* gene polymorphism reports, 73,004 participants had the info of 25-hydroxyvitamin D concentrations, and 6634 participants derived from the investigations of vitamin D intake.

Of these participants, 5272 HNC patients were enrolled, including 1,064 *VDR* gene polymorphism, 2,146 25-hydroxyvitamin D concentrations, and 2,062 vitamin D intake. Two cohort studies and a nested case–control study included only men. In most studies, participants were 40 years or older. Six studies were population-based, nine studies were hospital-based, and one study included health professionals. Most studies analyzed the risk of HNC by comparing the highest to the lowest fifth, fourth, or third of 25-hydroxyvitamin D or vitamin D intake. The *VDR* gene was genotyped by polymerase chain reaction-restriction fragment length polymorphism (PCR-RFLP) in selected studies. Radioimmunoassay, automated immunoassay, and chromatographic methods were used to detect the concentrations of 25-hydroxyvitamin D. All studies of vitamin D intake were investigated on food frequency questionnaires. **Table 1** summarized all the included studies, and **Supplementary Table 3** displayed the details of those studies.

**Table 1:** Summary characteristics of included studies.

Characteristics	Cohort studies	Case-control studies
Eligible studies		
No of studies (No of participants)	7 (71 034)	9 (10 874)
Follow-up at least three years	6 (70 803)	6 (9696)
Median (IQR) No of participants	522 (231–12 204)	1221 (289.5–2034.5)
Total No of HNC	1456	3816
Median (IQR) No of HNC	122 (51–398)	350 (140.5–736.5)
Median (IQR) % male	80 (48–100)	74 (68–75.5)
Country		
Europe	4 (22 624)	6 (8836)
North America	2 (48 322)	1 (1540)
Asian-Pacific	1 (88)	2 (498)
Vitamin D exposure		
*VDR* gene polymorphisms	—	4 (2270)
Vitamin D status	7 (71 034)	2 (1970)
Vitamin D intake	—	3 (6634)
Sample type		
Serum	5 (13 443)	1 (680)
Plasma	2 (57 591)	1 (1290)
Assay method		
PCR-RFLP	—	4 (2270)
Radioimmunoassay	2 (48 322)	—
Automated immunoassays	5 (16 566)	1 (680)
Chromatographic methods	1 (6146)	1(1290)
FFQ	—	3 (6634)
Baseline 25 hydroxyvitamin D		
<25	1 (47 800)	—
25–50	5 (58 308)	2 (1970)
50–75	4 (60 757)	—
>75	2 (47 888)	—
Outcome—No of studies (No of events) *		
HNC incidence	3 (217)	9 (3816)
HNC mortality	2 (349)	1 (145)
HNC overall survival	2 (118)	—

IQR, interquartile range.

*Several studies provided data on multiple outcomes of interest.

Values are number of studies (number of participants) unless stated otherwise.

A total of four publications reported the association between *VDR* gene polymorphism and the risk of HNC, all of which were case-control studies. Two studies focused on Asians, and the other two studies focused on Caucasians. The following four *VDR* SNPs were studied: *TaqI* (rs731236, alleles *t*/*T*), *FokI* (rs2228570, alleles *f*/*F*), and *BsmI* (rs1544410, alleles *B*/*b*). Three studies on *FokI* polymorphism included 1,000 patients and 1,119 controls, three studies on *TaqI* polymorphism included 893 patients and 1,030 controls, and two studies on *BsmI* polymorphism included 281 patients and 298 controls. Within the distribution of genotypes in the control groups, only 1 study deviated from HWE in the *BsmI* variant (*P* < 0.05). **Table 2** summarized the characteristics of these studies.

**Table 2:** Characteristics of case–control studies on *VDR* −*FokI* and −*TaqI* and −*BsmI* polymorphisms and cancer risk included in the meta-analysis.

Study	Location	Racial descent	Source of controls	Genotype distribution						*p* for HWE^a^	Genotyping method	Cancer location
				Case			Control					
				*F*/*F*	*F*/*f*	*f*/*f*	*F*/*F*	*F*/*f*	*f*/*f*			
Zeljic (44)	Serbia	Caucasians	Population-control	32	67	11	42	64	14	0.31	PCR-RFLP	Oral
Liu (43)	US	Caucasians	Population-control	293	330	96	293	381	147	0.23	PCR-RFLP	SCCHN
Huang (42)	China	Asian	Population-control	50	80	41	55	78	43	0.15	PCR-RFLP	NPC
				*T*/*T*	*T*/*t*	*t*/*t*	*T*/*T*	*T*/*t*	*t*/*t*			
Zeljic (44)	Serbia	Caucasians	Population-control	41	48	11	59	48	15	0.29	PCR-RFLP	Oral
Liu (43)	US	Caucasians	Population-control	256	360	103	271	396	154	0.66	PCR-RFLP	SCCHN
Bektas-Kayhan (41)	Turkey	Asian	Hospital-control	19	39	6	31	38	18	0.32	PCR-RFLP	OSCC
				*b*/*b*	*b*/*B*	*B*/*B*	*b*/*b*	*b*/*B*	*B*/*B*			
Zeljic (44)	Serbia	Caucasians	Population-control	39	71	0	59	60	3	0.01	PCR-RFLP	Oral
Huang (42)	China	Asian	Population-control	144	26	1	143	30	3	0.34	PCR-RFLP	NPC

^a^HWE, Hardy–Weinberg equilibrium in control.

### Quality Assessment

Eight articles had a low risk of bias (nine stars); the remaining eight articles had a moderate risk of bias (seven to eight stars). Newcastle-Ottawa Scale evaluated the selection of participants (12/16 of articles, 75%), comparability of study groups (15/16 of articles, 94%), and exposure (12/16 of articles, 75%). **Supplementary Tables 4, 5** and **Supplementary Figures 1, 2** showed the assessment of methodological quality. Most articles reported on the confounding factors, such as age, gender, smoking, and drinking. The majority of studies commonly used multivariable logistic regression models and Cox proportional hazard regression models to adjust these confounding factors (**Supplementary Table 6**). According to the GRADE summary of evidence, the quality of evidence was rated as very low to low for the incidence and outcomes of HNC on circulating 25-OHD, but very low for HNC incidence on vitamin D intake, and *VDR* gene polymorphism except for *FokI* gene polymorphism. **Supplementary Figures 3–7** showed the summary of findings for the GRADE assessment.

### Primary Outcome: HNC Incidence

**Figure 2:**
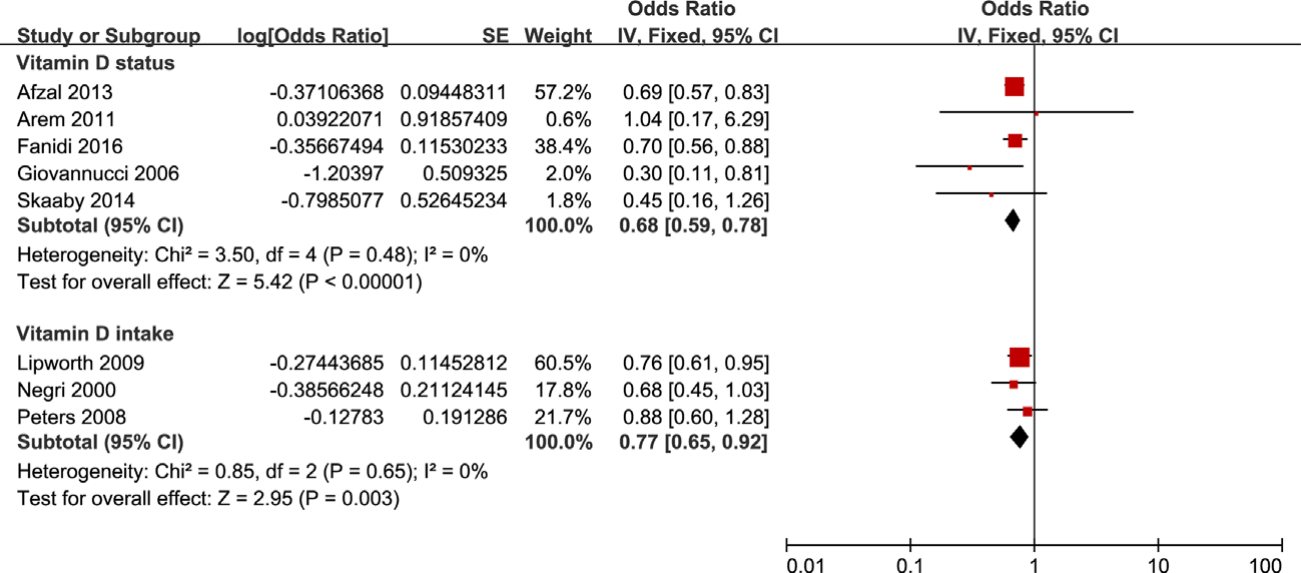
Forest plot of HNC incidence of studies evaluating vitamin D status and vitamin D intake. Using fixed effects models, for all observational studies, the estimates were 0.68(0.59 to 0.78) for vitamin D status; 0.77(0.65 to 0.92) for vitamin D intake.

Twelve studies that reported the proportions were pooled together to quantify the associations between vitamin D exposure and HNC incidence (four registered on *VDR* gene polymorphism, five on circulating 25-OHD, and three on vitamin D intake). Consequently, we found significant inverse associations between HNC incidence and vitamin D exposures, including a circulated concentration of 25-OHD and vitamin D intake (**Figure 2**). The pooled OR of top concentration levels of 25-OHD over the population with bottom levels, after the adjustment of potential confounding risk factors, was 0.68 (95% CI 0.59 to 0.78). As shown in **Supplementary Table 7**, similar conclusions were also obtained across other subgroup analyses.

As to vitamin D intake, three published studies contributed to the pooled results, in which one reported an inverse association, and the other two displayed null associations. The pooled OR was 0.77 (0.65 to 0.92) in a comparison of the highest to the lowest vitamin D intake category. Stratified analyses were summarized in **Supplementary Table 7**. The meta-analysis results for circulating concentration of 25-OHD and vitamin D intake were robust in sensitivity analyses.

**Figure 3:**
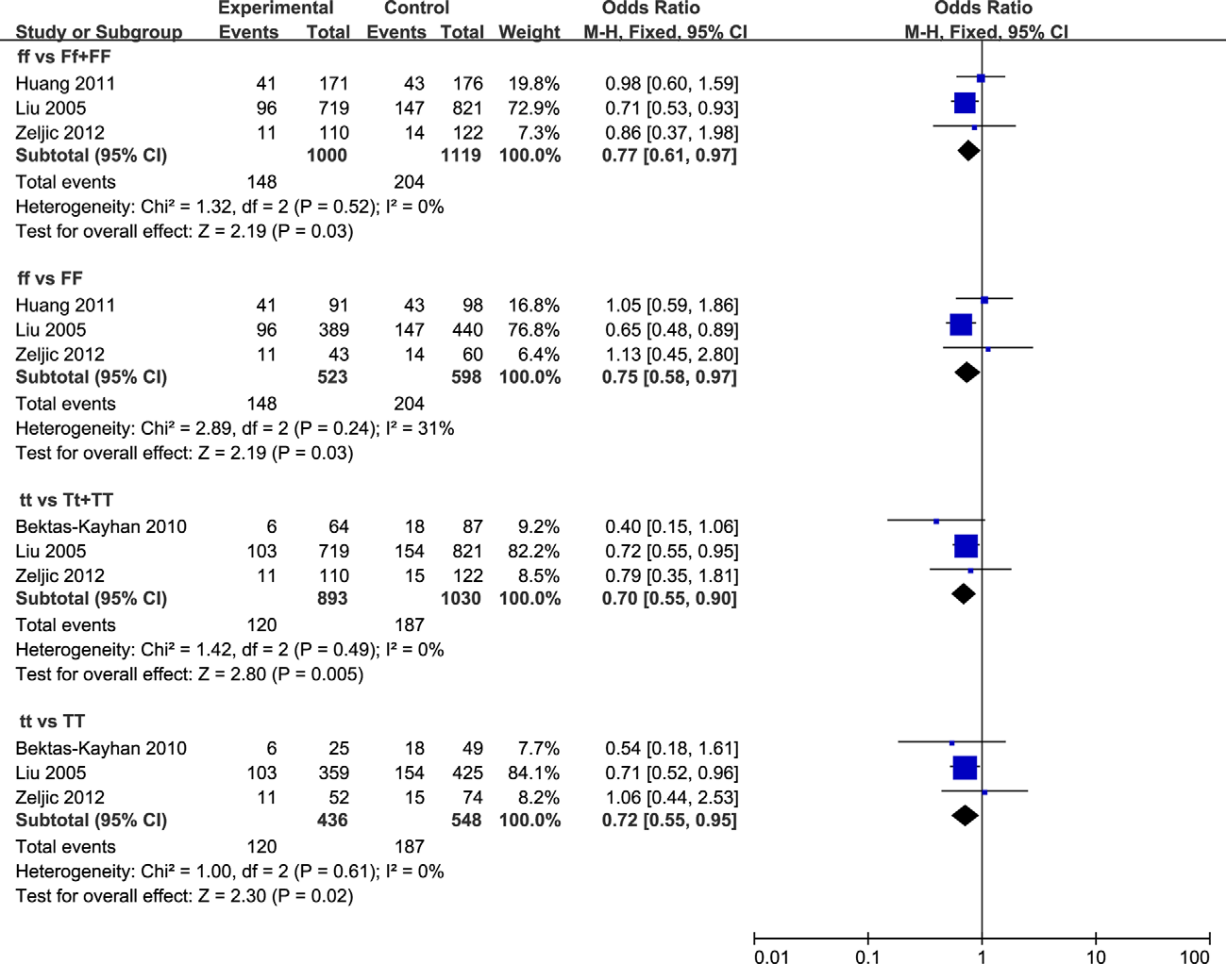
Forest plot of HNC incidence of studies evaluating *VDR* gene polymorphisms. Using fixed effects models, for all observational studies, the estimates were 0.77(0.61 to 0.97) for *ff* vs *Ff* + *FF*; 0.75(0.58 to 0.97) for *ff vs FF*; 0.70(0.55 to 0.90) for *tt vs Tt* + *TT*; 0.72(0.55 to 0.95) for *tt vs TT*.

A total of three relevant studies were examined for the association between the *FokI* polymorphism and HNC risk. The combined analyses revealed a significantly reduced risk of HNC incidence for this mutation in only two genetic models (*ff vs*. *Ff* + *FF*: OR = 0.77, 95% CI = 0.61 to 0.97, *I^2^* = 0%; *ff vs*. *FF*: OR = 0.75, 95% CI = 0.58 to 0.97, *I^2^* = 31%) (**Figure 3**). Subsequent analyses accounting for ethnicity revealed that a reduced HNC risk was observed in Caucasians for the recessive model (*ff vs*. *Ff* + *FF*: OR = 0.72, 95% CI = 0.55–0.94, *I^2^* = 0%). The subgroup analyses were reported in **Supplementary Table 8**.

Three studies were included in the analysis to determine whether *TaqI* polymorphism was associated with HNC risk. A significant reduction in HNC risk was observed in the overall population (*tt vs*. *Tt* + *TT*: OR = 0.70, 95% CI = 0.55 to 0.90, *I^2^* = 0%; *tt vs*. *TT*: OR = 0.72, 95% CI = 0.55 to 0.95, *I^2^* = 0%), as well as among Caucasian populations (*tt vs*. *Tt* + *TT*: OR = 0.73, 95% CI = 0.56 to 0.95, *I^2^* = 0%; *tt vs*. *TT*: OR = 0.74, 95% CI = 0.56 to 0.98, *I^2^* = 0%) (**Figure 3**). Furthermore, the stratified analyses were reported in **Supplementary Table 8**. There was one study performed by Bektas-Kayhan in relatively low quality. Sensitivity analyses by excluding this study did not change the pooled results.

Two studies were included in the analysis to determine whether *BsmI* polymorphism was associated with HNC risk. Overall, no significant associations were observed in all five models (**Supplementary Table 8**). Therefore, we did not perform the subgroup analysis to detect the association between HNC risk and *BsmI* mutation because too few studies were available to make a valid statistical test.

### Secondary Outcome: HNC Mortality

**Figure 4:**
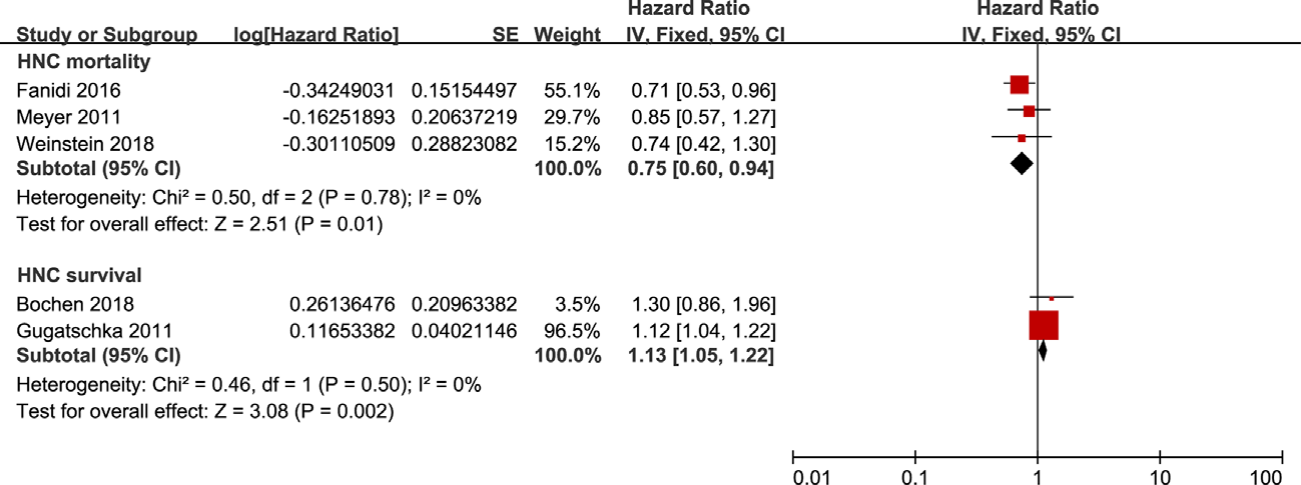
Forest plot of HNC mortality and HNC survival of studies evaluating vitamin D status. Using fixed effects models, for all observational studies, the estimates were 0.75(0.60 to 0.94) for HNC mortality; 1.13(1.05 to 1.22) for HNC survival.

Finally, we identified an inverse association between HNC mortality and 25-OHD levels, with an HR of 0.75 (95%CI 0.60 to 0.94) based on a fixed-effects model that pooled populations with an 8-12 years’ follow-up (**Figure 4**). To examine the robustness of the risk estimate, **Supplementary Table 9** displayed the results of the prespecified subgroup analyses. When performing the sensitivity analyses, including population-based studies for 25-OHD levels, the pooled HR for HNC mortality remained unchanged. Besides, the survival of HNC patients was significantly better in candidates with the highest circulating 25-OHD than that with the lowest circulating 25-OHD during a 4–5 years’ follow-up (**Figure 4**).

## Discussion

In this study, we comprehensively summarized the association between vitamin D exposures and HNC events across 16 individual reports. The primary findings indicate that elevated activities of vitamin D from diet intake, genomic polymorphisms, and circulated 25-OHD may protect people from HNC and improve the prognosis of HNC patients to some extent.

### Principal Findings and Comparison With Other Studies

Currently, vitamin D deficiency is a non-negligible health issue over the world (45); especially in the United States, 25 to 58% of adolescents and adults are deficient in vitamin D (46). Epidemiological studies suggest that vitamin D deficiency is associated with an increased incidence of cancer and worse outcomes. Vitamin D deficiency, as well as *VDR* knockout, enhances the development and growth of MC-26 colon and breast cancer xenografts in Balb/c mice (47, 48). A randomized trial breakthrough revealing the direct cancer risk reduction by vitamin D intake was reported by Lappe et al. (49). The purported health benefits of vitamin D are receiving increasing attention from the medical and public health professionals. To our best knowledge, this study is the first meta-analysis to investigate the association between HNC and vitamin D. Our meta-analysis of all available observational studies of vitamin D intake, suggests that vitamin D supplement may significantly reduce the risk of HNC incidence, which is consistent with previous studies (50–52). Vitamin D deficiency is highly prevalent among older, community-dwelling adults (53). Meantime, cancer also trends to be developed in the more ageing population, implying the potential opportunities for killing “two birds” with vitamin D supplementation in the older ones.

Dietary vitamin D could be converted into 25-OHD in the liver; this is the circulating form of vitamin D that is measured in the blood and clinically used to establish and monitor the vitamin D status. Substrate 25-OHD subsequently hydroxylated to form calcitriol by the cytochrome P450 enzyme CYP27B1 in the kidney. Activated calcitriol then binds to the *VDR* and regulates the expression of a diverse array of vitamin D responsive genes (54). Concerning HNC, a prospective cohort study assessing circulating 25-OHD levels in Finns found that the prevalence of vitamin D deficiency was higher in HNC patients; circulating 25-OHD in 65% of HNC patients was less than 50 nmol/L (55). This study indirectly supports our result that populations in a group with a high concentration of 25-OHD have a 32% lower incidence of HNC risk. Our results were similar with reports in breast and colorectal cancers, which also confirmed a beneficial role of 25-OHD levels in cancer prevention (56, 57). Furthermore, a prospective study in Asian populations reported that high circulating 25-OHD levels had a protective effect on the low risk of pan-cancer, but with the absence of any HNC cases (58). However, an investigation in prostate cancer does not support the negative association between 25-OHD and cancer risk (59), as we found in HNC, which may be attributed by variables in different cancer types and some unaccounted confounding such as lifestyle, and socioeconomic status absent in the studies.

Since the 5-year survival is a commonly used indicator for the prognosis of HNC, we include the HNC mortality in which patients were followed up for more than five years in our research, enabling to consolidate the reliability of our findings (60). Additionally, our study also determined the positive correlation of 25-OHD concentrations with HNC survival. As reported in a systematic review, insufficient circulating 25-OHD concentration increased cancer mortality (61). The consistent association of 25-OHD concentrations with cancer prognosis has been evaluated in subsequent meta-analyses (62, 63). A recent systematic review suggested an inverse relationship between the risk of HNC and 25(OH)D level and a direct relationship between 25(OH)D levels and overall HNC survival (36). We pooled independent but similar studies increases precision and therefore increases the confidence level of the findings and revealed trends that might not be apparent in a single study. Therefore, our findings on the risk and outcomes of HNC enlarge the scope of these conclusions, which also strengthens the notion of that circulating 25-OHD gifts prognosis and prevention benefit to diverse stable cancer patients, including HNC.

Except for ingestion, the genomic alteration analysis in our study shows a statistically significant causal relationship between the reduced risk of HNC incidence and *VDR FokI* polymorphism or the *TaqI* polymorphism. By pooling these results, the incidence of HNC was significantly lower in individuals with *ff* genotype than that of individuals with genotypes of *Ff* + *FF* or *FF*, and the incidence of HNC was significantly lower in individuals with *tt* genotype than that with genotypes of *Tt* + *TT* or *TT*. As mentioned previously, *VDR FokI*, and *BsmI* polymorphisms modulate the risk of breast cancer, skin cancer, and prostate cancer, which possibly affect cancer risk at any site of the body in Caucasians (64). Wang and colleagues have reported that *VDR TaqI* polymorphism is related to an increased risk of breast cancer, especially among Caucasian populations (65). In the meta-analysis published by Ntais (66), no significant associations are confirmed by the summary risk estimates, and no evidence that *BsmI* polymorphism modified the risk of prostate cancer is identified. However, an increase in cancer risk was also observed in candidates with *BsmI* polymorphism. In this report, the authors also found increased risks in oral, breast, and basal cell cancer, while the decreased risk in prostate cancer in *t* allele carriers of *TaqI* polymorphism; increased risks in ovarian and skin cancer, while the decreased risk in glioma in *f* allele carriers of *FokI* polymorphism (67). However, due to the small sample size and the limited number of studies examined, no significant association is observed between *BsmI* polymorphism and HNC risk in our analysis. The discrepancies may be explained by the distinct genetic backgrounds of cancer types and different functional mechanisms of vitamin D in multiple tissues. Mechanistically, 1,25(OH)_2_D_3_ influences gene transcription by binding to the promoter region of target genes, which also functions in a promoter-specific and cell-specific manner (68). The variation in specific DNA sequence, *VDR* isoforms, cell-specific phosphorylation, and co-regulators in different tissues could influence the binding capacity of the *VDR* to its target sequences. However, the underlying mechanisms of diverse *VDR* gene polymorphism in all human cancers remains to be further investigated.

Vitamin D deficiency is relatively feasible to HNC because patients usually suffer from chronic dysphagia and anorexia; Vitamin D deficiency is associated with a poor prognosis, peri- and intertumoral immune cell infiltration in cancers (26). An early phase human trial confirms a positive association between vitamin D treatment and the reduced infiltration of immune suppressive cells (69). Vitamin D supplementation increases the anti-tumor activity of NK cells and improves the prognosis through an anti-tumor immune response. Infiltration of both activated CD4^+^CD69^+^ T cells and regulatory Foxp3^+^CD4^+^ T cells into HNSCC tumor tissue contribute to prognosis (70). HNC Patients with higher 25(OH)D level also had higher levels of CD4^+^ T cell infiltration in the tumor and peritumor stroma and were associated with longer overall survival (34). Cytotoxic T lymphocytes (CTL) express both CYP27B1 and VDR, suggesting a coordinate regulation of VDR signaling pathway and CTL responses (71, 72). There is evidence suggesting that adequate vitamin D and VDR expression are required for T-cell antigen receptor signaling and subsequent T-cell activation (73). *In vitro* studies reveal an inhibitory effect of vitamin D on head and neck squamous cell carcinoma (HNSCC) cell proliferation, cell cycle as well as angiogenesis, associated with a higher sensitivity to chemotherapeutic agents (74). In an *in vivo* model, treatment with vitamin D delayed the carcinogenesis in the hamster buccal pouch (75). Therefore, it is reasonable to assume that vitamin D may reduce HNC incidence and mortality by its impacts on both the tumor cell and its surrounding immune cell types, which requires to be deeply clarified in the future.

### Strengths and Implications

In countries like the United States, the routine assessment of vitamin D status in old patients, and the recommendation of supplemental vitamin D have been increasing substantially for multiple reasons. Mitigating cancer incidence by vitamin D supplements has been advocated and validated in emerging studies. However, whether the benefit of vitamin D could cover the HNC incidence and mortality is unclear.

In our study, the inverse correlation of vitamin D and HNC incidence and mortality was confirmed in three angles (dietary/circulated/genomic levels), which involved in the metabolic process of vitamin D from nature to active substance. The cross-field validation in these three ways could consolidate our conclusions.

Moreover, one of our results is mainly driven by the subgroup analysis in population more than 50 years old, which will bring significant meaning to the community because HNC is highly prevalent around 50 years old. Based on data from almost 81,908 participants across 11 nations, the general ability of our findings is relatively solidified. Suppose this can be confirmed in future random clinical trials or more extensive population studies. In that case, a recommendation of vitamin D for people whose age is more than 50 may achieve enormous progress in preventing HNC rate worldwide. Undoubtedly, the appropriate quantity of supplemental vitamin D to avoid overdose-related side-effects needs further research.

### Limitations

We should be aware that available studies included in our study are observational investigations, which may lead to inevitable biases in the analysis. Observational reports of vitamin D intake cannot preclude the possibility that other confounding factors such as outdoor sun exposure and other physical conditions related to the bioavailability of 25-OHD may exist. Furthermore, these enrolled studies used food frequency questionnaires to assess dietary intake of vitamin D, which indicates that a potential bias from the inaccuracy induced by food frequency questionnaires. However, we can confirm the conclusion by the analysis from the circulating 25-OHD concentrations and *VDR* genomic phenotypes, which could counteract the drawbacks in food frequency questionnaires. The data composition is inadequate to conduct in-depth subgroup analysis, but the stratified and sensitivity analyses ensure that our results are relatively stable. Because of a few studies, we did not perform publication bias. All included literature was searched based on English; thus, language bias may also exist. The non-classical role of vitamin D signaling in stimulating innate immunity and suppressing inflammatory responses has been extensively explored, further research is needed on the direct effects of T lymphocytes in adaptive immunity.

### Conclusions

Elevated activities of vitamin D by diet intake, genomic polymorphisms, and circulated 25-OHD may protect people from HNC events and improve the prognosis of HNC patients. The finding that exposures to high 25-OHD level is more associated with the risk and outcomes of HNC, with evidence most consistent and effect sizes largest for incidence and mortality of HNC compared with vitamin D intake and *VDR* genomic polymorphisms. Further well-designed randomized controlled trials with larger sample sizes and different ethnic populations are required to clarify the present findings.

## Data Availability Statement

The original contributions presented in the study are included in the article/**Supplementary Material**. Further inquiries can be directed to the corresponding authors.

## Author Contributions

Conceptualization: YP, GZ, DH, YL, and XZ. Methodology: YP, GZ, YX, SZ, BT, and HH. Validation: YP, GZ, YX, SZ, BT, HH, and IW. Formal analysis: YP, GZ, and IW. Investigation: YP and GZ. Resources: IW, YL, and XZ. Data curation: YP, GZ, YX, SZ, BT, HH, and IW. Writing—original draft preparation: YP and GZ. Writing—review and editing: YX, SZ, BT, HH, and IW. Supervision: DH, YL, and XZ. Project administration: DH, YL, and XZ. Funding acquisition: DH, YL, and XZ. All authors contributed to the article and approved the submitted version.

## Funding

This work was supported by National Key Research and Development Project (Nos. 2020YFC1316900 and 2020YFC1316901), National Natural Science Foundation of China (Nos. 81974424, 81874133, 81772903, 81602389, and 82073009), Natural Science Foundation of Hunan Province (Nos. 2020JJ4827, 2019JJ50944, and 2018JJ2630), the Huxiang Young Talent Project (No. 2018RS3024), and the Project of Hunan Health Commission (B2019165).

## Conflict of Interest

The authors declare that the research was conducted in the absence of any commercial or financial relationships that could be construed as a potential conflict of interest.
