# Supplementary material for: Association Between Vitamin D Exposure and Head and Neck Cancer: A Systematic Review With Meta-Analysis
Source: Front Immunol. 2021 Feb 23;12:627226. doi: 10.3389/fimmu.2021.627226 (PMC7959800; doi:10.3389/fimmu.2021.627226)
Supplement: Supplementary file 1 [file DataSheet_1.docx]

Supplementary Material

# Supplementary Tables and Figures

## Supplementary Tables

**Supplementary Table 1.** Search strategy.

| **Database** | **Search strategy** |
| --- | --- |
| Pubmed | #1 “Head and Neck Neoplasms” [Mesh:NoExp] |
|  | #2 “Otorhinolaryngologic Neoplasms” [Mesh] OR “Neck Dissection” [Mesh] OR “Laryngectomy” [MeSH] OR HNSCC [tiab] OR SCCHN [tiab] OR OPSCC [tiab] OR OP-SCC [tiab] OR laryngectom* [tiab] OR (neck AND dissect*) [tiab] |
|  | #3 “Neoplasms” [Mesh] OR (cancer [tiab] OR cancers [tiab] OR cancerous [tiab] OR carcinoma* [tiab] OR neoplas* [tiab] OR tumor* [tiab] OR tumour* [tiab] OR malignan* [tiab] OR SCC [tiab]) |
|  | #4 “head and neck” [tiab] OR “head neck” [tiab] OR “head-neck” [tiab] OR “head-and-neck” [tiab] OR oral [tiab] OR oropharyn* [tiab] OR hypopharyn* [tiab] OR laryn* [tiab] OR nasopharyn* [tiab] mesopharyn* [tiab] OR pharyn* [tiab] OR tonsil* [tiab] OR “tongue base” [tiab] OR throat [tiab] OR mouth [tiab] |
|  | #5 #3 AND #4 |
|  | #6 #1 OR #2 OR #5 |
|  | #7 “Vitamin D” [Mesh] |
|  | #8 vit-d? [tiab] OR vitamin-d? [tiab] OR dihydroxyvitamin D? [tiab] OR dihydroxy-vitamin D? [tiab] OR hydroxyvitamin D? [tiab] OR hydroxyl-vitamin d? [tiab] OR dihydrotachysterol [tiab] OR colecalciferol [tiab] OR epicalcitriol [tiab] OR oxacalcitriol [tiab] OR alfacalcidol [tiab] OR calcifediolⅠ[tiab] OR calciferol? [tiab] OR calcipotriol [tiab] OR calcitriol [tiab] OR dihydroxycolecalciferol [tiab] OR hydroxycolecalciferol [tiab] OR seocalcitol [tiab] OR tacalcitol [tiab] OR oxavitamin [tiab] OR hydroxycholecalciferol? [tiab] OR calcidiol [tiab] OR calcipotriene [tiab] OR dihydroxycholecalciferol? [tiab] OR dihydroxy-cholecalciferol [tiab] OR cholecalciferol? [tiab] OR ergocalciferol? [tiab] OR epiergocalciferol [tiab] OR dihydroxyergocalciferol [tiab] OR dihydroxy-ergocalciferol [tiab] OR hydroxyl-ergocalciferol [tiab] OR hydroxyergocalciferol [tiab] OR doxercalciferol [tiab] OR hydroxycalciferol [tiab] OR hydroxyl-calciferol [tiab] OR dihydroxy-calciferol [tiab] OR dihydroxycalciferol [tiab] OR dihydrotachysterin [tiab] OR calcamine [tiab] OR ercalcidiol [tiab] |
|  | #9 #7 OR #8 |
|  | #10 #6 AND #9 |
| Embase | #1 exp “head and neck cancer”/ |
|  | #2 exp “neck dissection”/ |
|  | #3 exp laryngectomy/ |
|  | #4 HNSCC:ab,ti OR SCCHN:ab,ti OPSCC:ab,ti OP-SCC:ab,ti OR laryngectom*:ab,ti OR (neck and dissect*):ab,ti |
|  | #5 exp neoplasm/ |
|  | #6 cancer:ab,ti OR cancers:ab,ti OR cancerous:ab,ti OR carcinoma*:ab,ti OR neoplas*:ab,ti OR tumor*:ab,ti OR tumour*:ab,ti OR malignan*:ab,ti OR SCC:ab,ti |
|  | #7 #5 OR #6 |
|  | #8 (head and neck):ab,ti OR (head neck):ab,ti OR (head-neck):ab,ti OR (head-and-neck):ab,ti OR oral:ab,ti OR oropharyn*:ab,ti OR hypopharyn*:ab,ti OR laryn*:ab,ti OR nasopharyn*:ab,ti OR pharyn*:ab,ti OR mesopharyn*:ab,ti OR tonsil:ab,ti OR (tongue base):ab,ti OR throat:ab,ti OR mouth:ab,ti |
|  | #9 #7 AND #8 |
|  | #10 #1 OR #2 OR #3 OR #4 OR #9 |
|  | #11 exp “vitamin d”/ |
|  | #12 vitamin:ab,ti OR dihydroxyvitamin:ab,ti OR (dihydroxy-vitamin):ab,ti OR hydroxyvitamin:ab,ti OR (hydroxyl-vitamin):ab,ti |
|  | #13 d?:ab,ti |
|  | #14 #12 AND #13 |
|  | #15 vit-d?:ab,ti OR vitamin-d?:ab,ti OR dihydrotachysterol:ab,ti OR colecalciferol:ab,ti OR epicalcitriol:ab,ti OR oxacalcitriol:ab,ti OR alfacalcidol:ab,ti OR (calcifediolⅠ):ab,ti OR calciferol?:ab,ti OR calcipotriol:ab,ti OR calcitriol:ab,ti OR dihydroxycolecalciferol:ab,ti OR hydroxycolecalciferol:ab,ti OR seocalcitol:ab,ti OR tacalcitol:ab,ti OR oxavitamin:ab,ti OR hydroxycholecalciferol?:ab,ti OR calcidiol:ab,ti OR calcipotriene:ab,ti OR dihydroxycholecalciferol?:ab,ti OR dihydroxy-cholecalciferol:ab,ti OR cholecalciferol?:ab,ti OR ergocalciferol?:ab,ti OR (epiergocalcifero l):ab,ti OR dihydroxyergocalciferol:ab,ti OR dihydroxy-ergocalciferol:ab,ti OR hydroxyl-ergocalciferol:ab,ti OR hydroxyergocalciferol:ab,ti OR doxercalciferol:ab,ti OR hydroxycalciferol:ab,ti OR hydroxyl-calciferol:ab,ti OR dihydroxy-calciferol:ab,ti OR dihydroxycalciferol:ab,ti OR dihydrotachysterin:ab,ti OR calcamine:ab,ti OR ercalcidiol:ab,ti |
|  | #16 #11 OR #14 OR #15 |
|  | #17 #10 AND #16 |
| Web of science | #1 TS=(HNSCC OR SCCHN OR OPSCC OR OP-SCC OR laryngectom* OR (neck AND dissect*)) |
|  | #2 TS=(cancer* OR carcinoma* OR neoplas* OR tumor* OR tumour* OR malignan* OR SCC) |
|  | #3 TS=((head and neck) OR oral OR oropharyn* OR hypopharyn* OR laryn* OR nasopharyn* OR pharyn* OR mesopharyn* OR tonsil OR (tongue base) OR throat OR mouth) |
|  | #4 #2 AND #3 |
|  | #5 #1 OR #4 |
|  | #6 TS=(vitamin OR dihydroxyvitamin OR dihydroxy-vitamin OR hydroxyvitamin OR hydroxyl-vitamin) |
|  | #7 TS=(D OR D2 OR D3) |
|  | #8 #6 AND #7 |
|  | #9 TS=(vit-d? OR vitamin-d? OR dihydrotachysterol OR colecalciferol OR epicalcitriol OR oxacalcitriol OR alfacalcidol OR (calcifediol Ⅰ) OR calciferol? OR calcipotriol OR calcitriol OR dihydroxycolecalciferol OR hydroxycolecalciferol OR seocalcitol OR tacalcitol OR oxavitamin OR hydroxycholecalciferol? OR calcidiol OR calcipotriene OR dihydroxycholecalciferol? OR dihydroxy-cholecalciferol OR cholecalciferol? OR ergocalciferol? OR epiergocalciferol OR dihydroxyergocalciferol OR dihydroxy-ergocalciferol OR hydroxyl-ergocalciferol OR hydroxyergocalciferol OR doxercalciferol OR hydroxycalciferol OR hydroxyl-calciferol OR dihydroxy-calciferol OR dihydroxycalciferol OR dihydrotachysterin OR calcamine OR ercalcidiol) |
|  | #10 #8 OR #9 |
|  | #11 #5 AND #10 |
| Cochrane library | #1 MeSH descriptor Head and Neck Neoplasms, this term only |
|  | #2 MeSH descriptor Otorhinolaryngologic Neoplasms explode all trees |
|  | #3 MeSH descriptor Neck Dissection explode all trees |
|  | #4 MeSH descriptor Laryngectomy explode all trees |
|  | #5 MeSH descriptor Neoplasms explode all trees |
|  | #6 cancer*:ti,ab,kw OR carcinoma*:ti,ab,kw OR neoplas*:ti,ab,kw OR tumor*:ti,ab,kw OR tumour*:ti,ab,kw OR malignan*:ti,ab,kw OR SCC:ti,ab,kw |
|  | #7 “head and neck”:ti,ab,kw OR “head neck”:ti,ab,kw OR “head-neck”:ti,ab,kw OR “head-and-neck”:ti,ab,kw OR oral:ti,ab,kw OR oropharyn*:ti,ab,kw OR hypopharyn*:ti,ab,kw or laryn*:ti,ab,kw OR nasopharyn*:ti,ab,kw OR pharyn*:ti,ab,kw OR mesopharyn*:ti,ab,kw OR tonsil:ti,ab,kw OR “tongue base”:ti,ab,kw OR throat:ti,ab,kw OR mouth:ti,ab,kw |
|  | #8 (#5 OR #6) AND #7 |
|  | #9 HNSCC:ti,ab,kw OR SCCHN:ti,ab,kw OR OPSCC:ti,ab,kw OR OP-SCC:ti,ab,kw OR laryngectom*:ti,ab,kw OR (neck AND dissect*):ti,ab,kw |
|  | #10 #1 OR #2 OR #3 OR #4 OR #8 OR #9 |
|  | #11 MeSH descriptor vitamin D explode all trees |
|  | #12 vitamin:ti,ab,kw OR dihydroxyvitamin:ti,ab,kw OR dihydroxy-vitamin:ti,ab,kw OR hydroxyvitamin:ti,ab,kw OR hydroxyl-vitamin:ti,ab,kw |
|  | #13 d*:ti,ab,kw |
|  | #14 #12 AND #13 |
|  | #15 vit-d*:ti,ab,kw OR vitamin-d*:ti,ab,kw OR dihydrotachysterol:ti,ab,kw OR colecalciferol:ti,ab,kw OR epicalcitriol:ti,ab,kw OR oxacalcitriol:ti,ab,kw OR alfacalcidol:ti,ab,kw OR (calcifediolⅠ):ti,ab,kw OR calciferol*:ti,ab,kw OR calcipotriol:ti,ab,kw OR calcitriol:ti,ab,kw OR dihydroxycolecalciferol:ti,ab,kw OR hydroxycolecalciferol:ti,ab,kw OR seocalcitol:ti,ab,kw OR tacalcitol:ti,ab,kw OR oxavitamin:ti,ab,kw OR hydroxycholecalciferol*:ti,ab,kw OR calcidiol:ti,ab,kw OR calcipotriene:ti,ab,kw OR dihydroxycholecalciferol*:ti,ab,kw OR dihydroxy-cholecalciferol:ti,ab,kw OR cholecalciferol*:ti,ab,kw OR ergocalciferol*:ti,ab,kw OR (epiergocalcifero l):ti,ab,kw OR dihydroxyergocalciferol:ti,ab,kw OR dihydroxy-ergocalciferol:ti,ab,kw OR hydroxyl-ergocalciferol:ti,ab,kw OR hydroxyergocalciferol:ti,ab,kw OR doxercalciferol:ti,ab,kw OR hydroxycalciferol:ti,ab,kw OR hydroxyl-calciferol:ti,ab,kw OR dihydroxy-calciferol:ti,ab,kw OR dihydroxycalciferol:ti,ab,kw OR dihydrotachysterin:ti,ab,kw OR calcamine:ti,ab,kw OR ercalcidiol:ti,ab,kw |
|  | #16 #11 OR #14 OR #15 |
|  | #17 #10 AND #16 |
| ClinicalTrials.gov | #1 Condition= “head and neck neoplasms” OR “head and neck cancer” OR “head and neck squamous cell carcinoma” |
|  | #2 Other terms= vitamin D |
|  | #3 Study results= studies with results |
|  | #4 Status= completed |
| WHO International Clinical Trials Registry Platform (ICTRP) | #1 Condition= head and neck neoplasms OR head and neck cancer OR head and neck squamous cell carcinoma |
|  | #2 Intervention= vitamin D |

**Supplementary Table 2.** List of excluded articles.

| ID | AUTHOR/YEAR | TITLE | REASONS |
| --- | --- | --- | --- |
| 1 | Abboud 2008 | Is therapy with calcium and vitamin D and parathyroid autotransplantation useful in total thyroidectomy for preventing hypocalcemia? | Not adults diagnosed with HNC |
| 2 | Antonoglou 2015 | Associations Between Serum 25-Hydroxyvitamin D and Periodontal Pocketing and Gingival Bleeding: Results of a Study in a Non-Smoking Population in Finland | Not adults diagnosed with HNC |
| 3 | Arthur 2007 | Vitamin D | Not adults diagnosed with HNC |
| 4 | Azuma 1989 | Induction of cells with a chondrocyte-like phenotype by treatment with 1α,25-dihydroxyvitamin D3 in a human salivary acinar cell line | Not adults diagnosed with HNC |
| 5 | Bahramian 2018 | Comparing Serum and Salivary Levels of Vitamin D in Patients with Recurrent Aphthous Stomatitis and Healthy Individuals | Not adults diagnosed with HNC |
| 6 | Beer 2005 | Pharmacokinetics and tolerability of a single dose of DN-101, a new formulation of calcitriol, in patients with cancer | Not adults diagnosed with HNC |
| 7 | Beer 2007 | Phase I study of weekly DN-101, a new formulation of calcitriol, in patients with cancer | Not adults diagnosed with HNC |
| 8 | Bellantone 2002 | Is routine supplementation therapy (calcium and vitamin D) useful after total thyroidectomy? | Not adults diagnosed with HNC |
| 9 | Blum 2008 | Body size and serum 25 hydroxy vitamin D response to oral supplements in healthy older adults | Not adults diagnosed with HNC |
| 10 | Brake 2011 | Complementary and alternative medicine use in the thyroid patients of a head and neck practice | Not adults diagnosed with HNC |
| 11 | Carr 2014 | A single parathyroid hormone level obtained 4 hours after total thyroidectomy predicts the need for postoperative calcium supplementation | Not adults diagnosed with HNC |
| 12 | Cayo 2012 | Predicting the need for calcium and calcitriol supplementation after total thyroidectomy: Results of a prospective, randomized study | Not adults diagnosed with HNC |
| 13 | Chabrol 2020 | Randomized clinical trials of oral vitamin D supplementation in need of a paradigm change: The vitamin D autacoid paradigm | Not adults diagnosed with HNC |
| 14 | Docimo 2013 | Total thyroidectomy without prophylactic central neck dissection combined with routine oral calcium and vitamin d supplements: Is it a good option to achieve a low recurrence rate avoiding hypocalcemia? a retrospective study | Not adults diagnosed with HNC |
| 15 | Euctr 2009 | Effects of vitamin D supplementation in healthy women and men on immunological, endocrine and metabolic parameters | Not adults diagnosed with HNC |
| 16 | Euctr 2013 | The effect on quality of life of Vitamin D administration for advanced cancer treatment | Not adults diagnosed with HNC |
| 17 | Euctr 2012 | Feasibility study for a larger randomised trial of the effect of taking vitamin D supplements on health and lifespan in men and women aged 65 to 84 | Not adults diagnosed with HNC |
| 18 | Euctr 2017 | Vitamin D supplementation for patients with terminal cancer diagnosis - The patients will receive randomly either Vitamin D or inactive substance (placebo). Neither the patients or study personnel will know what treatment the patients will receive | Not adults diagnosed with HNC |
| 19 | Garland 2011 | Vitamin D supplement doses and serum 25-Hydroxyvitamin D in the range associated with cancer prevention | Not adults diagnosed with HNC |
| 20 | Grant 2009 | In defense of the sun: An estimate of changes in mortality rates in the United States if mean serum 25-hydroxyvitamin D levels were raised to 45 ng/mL by solar ultraviolet-B irradiance | Not adults diagnosed with HNC |
| 21 | Grant 2011 | Health benefit of increased serum 25(OH)D levels from oral intake and ultraviolet-B irradiance in the Nordic countries | Not adults diagnosed with HNC |
| 22 | Grant 2010 | Health benefits of higher serum 25-hydroxyvitamin D levels in The Netherlands | Not adults diagnosed with HNC |
| 23 | Guerrieri 2013 | Vitamin D and overall mortality | Not adults diagnosed with HNC |
| 24 | Gupta 2006 | Hypercalcemia due to vitamin D-secreting hodgkin's lymphoma exacerbated by oral calcium supplementation | Not adults diagnosed with HNC |
| 25 | Harvie 2014 | Nutritional supplements and cancer: potential benefits and proven harms | Not adults diagnosed with HNC |
| 26 | Heaney 2008 | Vitamin D in health and disease | Not adults diagnosed with HNC |
| 27 | Juzeniene 2012 | Malignant melanomas on head/neck and foot: differences in time and latitudinal trends in Norway | Not adults diagnosed with HNC |
| 28 | Kerr 1990 | Maintenance of serum calcium after total thyroparathyroidectomy | Not adults diagnosed with HNC |
| 29 | Krzyscin 2016 | Optimal vitamin D-3 daily intake of 2000 IU inferred from modeled solar exposure of ancestral humans in Northern Tanzania | Not adults diagnosed with HNC |
| 30 | Kujundzic 2016 | Association of vdr, cyp27b1, cyp24a1 and mthfr gene polymorphisms with oral lichen planus risk | Not adults diagnosed with HNC |
| 31 | McCullough 2019 | Daily oral dosing of vitamin D3 using 5000 TO 50,000 international units a day in long-term hospitalized patients: Insights from a seven year experience | Not adults diagnosed with HNC |
| 32 | Mitra 2011 | Effect of central compartment neck dissection on hypocalcaemia incidence after total thyroidectomy for carcinoma | Not adults diagnosed with HNC |
| 33 | Moore 1994 | Oral calcium supplements to enhance early hospital discharge after bilateral surgical treatment of the thyroid gland or exploration of the parathyroid glands | Not adults diagnosed with HNC |
| 34 | Mucke 2017 | Supportive therapy part 2. Micronutrients | Not adults diagnosed with HNC |
| 35 | Nct 2010 | Pilot Pharmacokinetic Study of Daily Versus Monthly High-Dose Cholecalciferol Supplementation | Not adults diagnosed with HNC |
| 36 | Nct 2010 | Vitamin D Dose Finding Study | Not adults diagnosed with HNC |
| 37 | Nct 2010 | Vitamin D and Omega-3 Trial (VITAL) | Not adults diagnosed with HNC |
| 38 | Nct 2017 | 'Palliative-D' Vitamin D to Palliative Cancer Patients | Not adults diagnosed with HNC |
| 39 | Nct 2018 | Vitamin D as a Nutritional Neoadjuvant During Photodynamic Therapy of Basal Cell Carcinoma | Not adults diagnosed with HNC |
| 40 | Nct 2018 | Vitamin D and Photodynamic Therapy for Treatment of BCC in BCNS | Not adults diagnosed with HNC |
| 41 | Neale 2016 | The D-Health Trial: A randomized trial of vitamin D for prevention of mortality and cancer | Not adults diagnosed with HNC |
| 42 | Omura 2016 | Optimal Dose of Vitamin D3 400 I.U. for Average Adults has A Significant Anti-Cancer Effect, While Widely Used 2000 I.U. or Higher Promotes Cancer: Marked Reduction of Taurine & 1α, 25(OH)2D3 Was Found In Various Cancer Tissues and Oral Intake of Optimal Dose of Taurine 175mg for Average Adults, Rather Than 500mg, Was Found to Be A New Potentially Safe and More Effective Method of Cancer Treatment | Not adults diagnosed with HNC |
| 43 | Oteri 2016 | Does Vitamin D3 Have an Impact on Clinical and Biochemical Parameters Related to Third Molar Surgery | Not adults diagnosed with HNC |
| 44 | Proye 1990 | The parathyroid risk in thyroid surgery. Argument against the early postoperative prescription of vitamin D. Experience with 729 thyroidectomies in 1988 | Not adults diagnosed with HNC |
| 45 | Puzziello 2014 | Hypocalcemia following thyroid surgery: incidence and risk factors. A longitudinal multicenter study comprising 2,631 patients | Not adults diagnosed with HNC |
| 46 | Roh 2006 | Routine oral calcium and vitamin D supplements for prevention of hypocalcemia after total thyroidectomy | Not adults diagnosed with HNC |
| 47 | Salle 2012 | Vitamin D: status, daily requirements and role in extra-osseous processes | Not adults diagnosed with HNC |
| 48 | Saulenas 1988 | Vitamin D and retinoblastoma. The presence of receptors and inhibition of growth in vitro | Not adults diagnosed with HNC |
| 49 | Shirazi 2013 | Determinants of serum levels of vitamin D: A study of life-style, menopausal status, dietary intake, serum calcium, and PTH | Not adults diagnosed with HNC |
| 50 | Stewart 2005 | 25-hydroxyvitamin D deficiency is a risk factor for symptoms of postoperative hypocalcemia and secondary hyperparathyroidism after minimally invasive parathyroidectomy | Not adults diagnosed with HNC |
| 51 | Terushkin 2010 | Estimated equivalency of vitamin D production from natural sun exposure versus oral vitamin D supplementation across seasons at two US latitudes | Not adults diagnosed with HNC |
| 52 | Tuohimaa 2007 | Does solar exposure, as indicated by the non-melanoma skin cancers, protect from solid cancers: Vitamin D as a possible explanation | Not adults diagnosed with HNC |
| 53 | Vashi 2010 | Impact of oral vitamin D supplementation on serum 25-hydroxyvitamin D levels in oncology | Not adults diagnosed with HNC |
| 54 | Tavera 2006 | Convergence of vitamin D and retinoic acid signalling at a common hormone response element | Not adults diagnosed with HNC |
| 55 | Vieth 2007 | Vitamin D toxicity, policy, and science | Not adults diagnosed with HNC |
| 56 | Akutsu 2001 | Amphiregulin is a vitamin D-3 target gene in squamous cell and breast carcinoma | No measurement of relevant vitamin D exposures |
| 57 | Alagbala 2006 | Antitumor effects of two less-calcemic vitamin D analogs (paricalcitol and QW-1624F(2)-2) in squamous cell carcinoma cells | No measurement of relevant vitamin D exposures |
| 58 | Bairati 2005 | Randomized trial of antioxidant vitamins to prevent acute adverse effects of radiation therapy in head and neck cancer patients | No measurement of relevant vitamin D exposures |
| 59 | Cheng 2007 | Carotenoids suppress proliferating cell nuclear antigen and cyclin D-1 expression in oral carcinogenic models | No measurement of relevant vitamin D exposures |
| 60 | Houlton 2011 | PACU PTH facilitates safe outpatient total thyroidectomy | No measurement of relevant vitamin D exposures |
| 61 | Lee 2007 | Subcellular localisation of BAG-1 and its regulation of vitamin D receptor-mediated transactivation and involucrin expression in oral keratinocytes: implications for oral carcinogenesis | No measurement of relevant vitamin D exposures |
| 62 | Nct 2005 | Beta-carotene and Alpha-tocopherol Chemoprevention of Second Primary Malignancies in Head and Neck Cancer Patients | No measurement of relevant vitamin D exposures |
| 63 | Orell 2017 | Cachexia at diagnosis is associated with poor survival in head and neck cancer patients | No measurement of relevant vitamin D exposures |
| 64 | Pavitt 2007 | Interventions for the treatment of oral and oropharyngeal cancers: Immunotherapy/biotherapy | No measurement of relevant vitamin D exposures |
| 65 | Randhawa 2015 | Integrated network analysis and logistic regression modeling identify stage-specific genes in Oral Squamous Cell Carcinoma | No measurement of relevant vitamin D exposures |
| 66 | Rougereau 1987 | Fat soluble vitamins and cancer localization associated to an abnormal ketone derivative of D3 vitamin: carcinomedin | No measurement of relevant vitamin D exposures |
| 67 | Shaikh 2014 | Clinico-pathological pattern of thyroid disease treated surgically | No measurement of relevant vitamin D exposures |
| 68 | Skaaby 2014 | Filaggrin loss-of-function mutations and incident cancer: a population-based study | No measurement of relevant vitamin D exposures |
| 69 | Wieder 2003 | Pharmacokinetics and safety of ILX23-7553, a non-calcemic-vitamin D 3 analogue, in a phase I study of patients with advanced malignancies | No measurement of relevant vitamin D exposures |
| 70 | Fang 2017 | Risk factors associated with oral and maxillofacial benign tumors: A case-control study | No measurement of relevant vitamin D exposures |
| 71 | Zhang 2015 | In serum, higher parathyroid hormone but not lower vitamin d is associated with oral squamous cell carcinoma | Associations of relevant vitamin D with HNC not reported/not derivable from reported data |
| 72 | Lathers 2001 | Phase IB study of 25-hydroxyvitamin d3 treatment to diminish suppressor cells in head and neck cancer patients | Associations of relevant vitamin D with HNC not reported/not derivable from reported data |
| 73 | Kulbersh 2009 | 1α,25-Dihydroxyvitamin D3 to skew intratumoral levels of immune inhibitory CD34+ progenitor cells into dendritic cells | Associations of relevant vitamin D with HNC not reported/not derivable from reported data |
| 74 | Dudding 2018 | Assessing the causal association between 25-hydroxyvitamin D and the risk of oral and oropharyngeal cancer using Mendelian randomization | Associations of relevant vitamin D with HNC not reported/not derivable from reported data |
| 75 | Deng 2020 | Low serum 25-hydroxyvitamin D3 levels and late delayed radiation-induced brain injury in patients with nasopharyngeal carcinoma: A case–control study | Associations of relevant vitamin D with HNC not reported/not derivable from reported data |
| 76 | Anand 2017 | Expression of Vitamin D receptor and Vitamin D status in patients with oral neoplasms and effect of Vitamin D supplementation on quality of life in advanced cancer treatment | Associations of relevant vitamin D with HNC not reported/not derivable from reported data |
| 77 | Young 2015 | Cytokine and adipokine levels in patients with premalignant oral lesions or in patients with oral cancer who did or did not receive 1α,25-dihydroxyvitamin d<inf>3</inf> treatment upon cancer diagnosis | Associations of relevant vitamin D with HNC not reported/not derivable from reported data |
| 78 | Wang 2011 | To Supplement or Not to Supplement: A Cost-Utility Analysis of Calcium and Vitamin D Repletion in Patients After Thyroidectomy | Associations of relevant vitamin D with HNC not reported/not derivable from reported data |
| 79 | Mai 2020 | Solar Ultraviolet Radiation and Vitamin D Deficiency on Epstein-Barr Virus Reactivation: Observational and Genetic Evidence From a Nasopharyngeal Carcinoma- Endemic Population | Associations of relevant vitamin D with HNC not reported/not derivable from reported data |
| 80 | Walker 2012 | Immunological modulation by 1α,25-dihydroxyvitamin D3 in patients with squamous cell carcinoma of the head and neck | Associations of relevant vitamin D with HNC not reported/not derivable from reported data |
| 81 | Vyas 2010 | Complementary and alternative medicine use in patients presenting to a head and neck oncology clinic | Associations of relevant vitamin D with HNC not reported/not derivable from reported data |
| 82 | Nejatinamin 2018 | Head and Neck Cancer Patients Do Not Meet Recommended Intakes of Micronutrients without Consuming Fortified Products | Associations of relevant vitamin D with HNC not reported/not derivable from reported data |
| 83 | Nejatinamini 2018 | Poor vitamin status is associated with skeletal muscle loss and mucositis in head and neck cancer patients | Associations of relevant vitamin D with HNC not reported/not derivable from reported data |
| 84 | Lathers 2004 | Phase 1B study to improve immune responses in head and neck cancer patients using escalating doses of 25-hydroxyvitamm D3 | Associations of relevant vitamin D with HNC not reported/not derivable from reported data |
| 85 | Grimm 2015 | Serum vitamin D levels of patients with oral squamous cell carcinoma (OSCC) and expression of vitamin D receptor in oral precancerous lesions and OSCC | Associations of relevant vitamin D with HNC not reported/not derivable from reported data |
| 86 | Walsh 2010 | Use of alpha,25-dihydroxyvitamin D3 treatment to stimulate immune infiltration into head and neck squamous cell carcinoma | Not observational studies |
| 87 | Scragg 2018 | Monthly High-Dose Vitamin D Supplementation and Cancer Risk: A Post Hoc Analysis of the Vitamin D Assessment Randomized Clinical Trial | Not observational studies |
| 88 | Jprn 2009 | A randomized, double blind, comparative study of vitamin D3 versus placebo in patients with head and neck squamous carcinoma to prevent relapse after operation | Not observational studies |
| 89 | Rosen 2013 | Vitamin D levels differ by cancer diagnosis and decline over time in survivors of childhood cancer | Children |
| 90 | Mulligan 2012 | Vitamin D3 deficiency increases sinus mucosa dendritic cells in pediatric chronic rhinosinusitis with nasal polyps | Children |
| 91 | Fouda 2018 | Hypovitamininosis D in Childhood Cancer Survivors: Importance of Vitamin D Supplementation and Measurement over Different Points of Time | Children |
| 92 | Zhao 2019 | Vitamin D/VDR signaling suppresses microRNA-802-induced apoptosis of keratinocytes in oral lichen planus | Non-human studies |
| 93 | Yuan 2014 | Vitamin D signaling regulates oral keratinocyte proliferation in vitro and in vivo | Non-human studies |
| 94 | Vincent 2019 | Preclinical Prevention Trial of Calcitriol: Impact of Stage of Intervention and Duration of Treatment on Oral Carcinogenesis | Non-human studies |
| 95 | Verma 2020 | Impact of dietary vitamin D on initiation and progression of oral cancer | Non-human studies |
| 96 | Prudencio 2001 | Action of low calcemic 1 alpha,25-dihydroxyvitamin D-3 analogue EB1089 in head and neck squamous cell carcinoma | Non-human studies |
| 97 | Jiang 2015 | Inhibition of 4-nitroquinoline-1-oxide-induced oral carcinogenesis by dietary calcium | Non-human studies |
| 98 | Gándara 2012 | Synthesis and biological evaluation of a new vitamin D2 analogue | Non-human studies |
| 99 | Bothwell 2015 | Impact of short-term 1,25-dihydroxyvitamin D<inf>3</inf> on the chemopreventive efficacy of erlotinib against oral cancer | Non-human studies |
| 100 | Binderup 1988 | Effects of a novel vitamin D analogue MC903 on cell proliferation and differentiation in vitro and on calcium metabolism in vivo | Non-human studies |
| 101 | Hager 2001 | 1,25(OH)2 vitamin D3 induces elevated expression of the cell cycle-regulating genes p21 and p27 in squamous carcinoma cell lines of the head and neck | Non-human studies |
| 102 | Kornfehl 1996 | Antiproliferative effects of the biologically active metabolite of vitamin D3 (1,25 [OH]2 D3) on head and neck squamous cell carcinoma cell lines | Non-human studies |
| 103 | Hager 2004 | Molecular Analysis of p21 Promoter Activity Isolated from Squamous Carcinoma Cell Lines of the Head and Neck under the Influence of 1,25(OH) 2 Vitamin D3 and its Analogs | Non-human studies |
| 104 | Hussein 2019 | 1,25-Dihydroxyvitamin D3 inhibits oral squamous cell carcinoma cell growth via microRNA regulation | Non-human studies |
| 105 | Lu 2009 | Inhibitory effect of 1,25(OH)2D3 on proliferation of human laryngeal carcinoma cells and potential mechanisms | Non-human studies |
| 106 | Lu 2008 | Vitamin D3 analogue EB1089 inhibits the proliferation of human laryngeal squamous carcinoma cells via p57 | Non-human studies |
| 107 | Satake 2003 | Anti-tumor effect of vitamin A and D on head and neck squamous cell carcinoma | Non-human studies |
| 108 | Grimm 2013 | Is 1,25-dihydroxyvitamin D3 receptor expression a potential Achilles' heel of CD44+ oral squamous cell carcinoma cells? | Non-human studies |
| 109 | Grimm 2017 | Erratum to: Is 1,25-dihydroxyvitamin D3 receptor expression a potential Achilles’ heel of CD44+ oral squamous cell carcinoma cells? | Non-human studies |
| 110 | Zhao 2017 | Knockdown of Snail inhibits epithelial-mesenchymal transition of human laryngeal squamous cell carcinoma Hep-2 cells through the vitamin D receptor signaling pathway | Non-human studies |
| 111 | Cai 2006 | In vitro inhibition of human laryngeal squamous cell carcinoma cell line by 1,25(OH)2D3 | Non-human studies |
| 112 | Osafi 2014 | Differential effects of 1,25-dihydroxyvitamin D₃ on oral squamous cell carcinomas in vitro | Non-human studies |
| 113 | Ferronato 2019 | Synthesis of a novel analog of calcitriol and its biological evaluation as antitumor agent | Non-human studies |
| 114 | Lin 2002 | Expression profiling in squamous carcinoma cells reveals pleiotropic effects of vitamin D-3 analog EB1089 signaling on cell proliferation, differentiation, and immune system regulation | Non-human studies |
| 115 | Chiang 2013 | MART-10, a novel vitamin D analog, inhibits head and neck squamous carcinoma cells growth through cell cycle arrest at G0/G1 with upregulation of p21 and p27 and downregulation of telomerase | Non-human studies |
| 116 | Wietrzyk 2007 | The effect of combined treatment on head and neck human cancer cell lines with novel analogs of calcitriol and cytostatics | Non-human studies |
| 117 | Yang 2016 | MART-10, a newly synthesized vitamin D analog, represses metastatic potential of head and neck squamous carcinoma cells | Non-human studies |
| 118 | Sundaram 2014 | 1α,25-Dihydroxyvitamin D3 Modulates CYP2R1 Gene Expression in Human Oral Squamous Cell Carcinoma Tumor Cells | Non-human studies |
| 119 | Abe 1998 | Inhibitory effect of 1,25-dihydroxyvitamin D3 and 9-cis-retinoic acid on parathyroid hormone-related protein expression by oral cancer cells (HSC- 3) | Non-human studies |
| 120 | Akutsu 2001 | Regulation of gene expression by 1α,25-dihydroxyvitamin D3 and its analog EB1089 under growth-inhibitory conditions in squamous carcinoma cells | Non-human studies |
| 121 | Dalirsani 2012 | The effects of 5-fluorouracil alone and in combination with 13-cis retinoic acid and vitamin D3 on human oral squamous cell carcinoma lines | Non-human studies |
| 122 | Enepekides 1999 | The independent and combined effects of RAR-, RXR-, and VDR-selective ligands on the growth of squamous cell carcinoma in vitro | Non-human studies |
| 123 | Gedlicka 2006 | 1,25(OH)2Vitamin D3 induces elevated expression of the cell cycle inhibitor p18 in a squamous cell carcinoma cell line of the head and neck | Non-human studies |
| 124 | Huang 2016 | 1,25-Dihydroxyvitamin D3 alleviates salivary adenoid cystic carcinoma progression by suppressing GPX1 expression through the NF-κB pathway | Non-human studies |
| 125 | Huang 2019 | Vitamin D promotes the cisplatin sensitivity of oral squamous cell carcinoma by inhibiting LCN2-modulated NF-κB pathway activation through RPS3 | Non-human studies |
| 126 | Konya 2018 | Vitamin D downregulates the IL-23 receptor pathway in human mucosal group 3 innate lymphoid cells | Non-human studies |
| 127 | Rosli 2014 | 1 alpha,25(OH)(2)D-3 inhibits FGF-2 release from oral squamous cell carcinoma cells through down-regulation of HBp17/FGFBP-1 | Non-human studies |
| 128 | Sato 1997 | Emergence of osteoblast-like cells in a neoplastic human salivary cancer cell line after treatment with 22-oxa-1α, 25-dihydroxyvitamin D3 | Non-human studies |
| 129 | Schmiedlin 1997 | Expression of enzymatically active CYP3A4 by Caco-2 cells grown on extracellular matrix-coated permeable supports in the presence of 1α,25- dihydroxyvitamin D3 | Non-human studies |
| 130 | Shintani 2017 | Eldecalcitol (ED-71), an analog of 1α,25(OH)2D3, inhibits the growth of squamous cell carcinoma (SCC) cells in vitro and in vivo by down-regulating expression of heparin-binding protein 17/fibroblast growth factor-binding protein-1 (HBp17/FGFBP-1) and FGF-2 | Non-human studies |
| 131 | Akita 2006 | Enhancement of the photodynamic effects on human orat squamous cell carcinoma cell lines by treatment with calcipotriol | Non-human studies |
| 132 | Verma 2020 | Impact of dietary vitamin D on initiation and progression of oral cancer | Non-human studies |
| 133 | Wang 2017 | Association of vitamin D receptor and its genetic polymorphisms with nasopharyngeal carcinoma | Articles written in non-English |
| 134 | Ringe 2011 | Vitamin D deficiency in Germany, is it a danger for increased morbidityand mortality? | Articles written in non-English |
| 135 | Rougereau 1993 | Application of multifactorial statistical calculation in research for a correlation between lipid soluble vitamins and cancer | Articles written in non-English |
| 136 | Tsukuda 1998 | Influence of treatment with differentiation-inducing drugs on head and neck carcinoma cell lines | Articles written in non-English |
| 137 | Calmarza 2018 | Vitamin D levels in patients with recent cancer diagnosis | Articles written in non-English |
| 138 | Szabó 2011 | Skeletal and extra-skeletal consequences of vitamin D defi ciency | Articles written in non-English |
| 139 | Gandini 2014 | Vitamin D receptor polymorphisms and cancer | Review |
| 140 | Zhang 2010 | Vitamin D in health and disease: Current perspectives | Review |
| 141 | Sanders 2013 | Is High Dose Vitamin D Harmful? | Review |
| 142 | Huang 2020 | The association between vitamin deficiency and otolaryngologic diseases: A therapeutic target | Review |
| 143 | Grant 2011 | Requirements for vitamin D across the life span | Review |
| 144 | Oshiro 2013 | Determining a natural vitamin D level The evidence for a 25(OH)D level within the range of 40-60 ng/mL as the new normal | Review |
| 145 | Rautiainen 2016 | Dietary supplements and disease prevention - a global overview | Review |
| 146 | Ravasco 2019 | Nutrition in Cancer Patients | Review |
| 147 | Nagpal 2001 | Vitamin D analogs: Mechanism of action and therapeutic applications | Review |
| 148 | Chen 2010 | Relationship between cancer mortality/incidence and ambient ultraviolet B irradiance in China | Ecologic studies |
| 149 | Boscoe 2006 | Solar ultraviolet-B exposure and cancer incidence and mortality in the United States, 1993-2002 | Ecologic studies |
| 150 | Huang 2002 | Antiproliferative and radiation-enhancing effects of vitamin D analog, paricalcitol, on head and neck squamous cell carcinoma | Conference articles |
| 151 | Linxweiler 2019 | Vitamin D in head and neck cancer | Conference articles |
| 152 | Mocanu 2008 | Vitamin D Deficiency and Oxidative Stress in Patients with Oral Squamous Cell Carcinoma and Healthy Subjects | Conference articles |
| 153 | Oak 2019 | Novel noncalcemic vitamin D hydroxyderivatives downregulate SHH and Wnt signaling pathways and inhibit spheroid formation in human oral squamous cell carcinoma and murine basal cell carcinoma | Conference articles |
| 154 | Orell 2012 | High prevalence of vitamin D insufficiency in patients with head and neck cancer at diagnosis | Insufficient data on studies |
| 155 | Hama 2011 | Prognostic Significance of Vitamin D Receptor Polymorphisms in Head and Neck Squamous Cell Carcinoma | Insufficient data on studies |
| 156 | Azad 2013 | Genetic sequence variants in vitamin D metabolism pathway genes, serum vitamin D level and outcome in head and neck cancer patients | Insufficient data on studies |
| 157 | Deschasaux 2015 | Prospective associations between Vitamin D status, Vitamin D-related gene polymorphisms, and risk of tobacco-related cancers | Insufficient data on studies |
| 158 | Freedman 2010 | Serum 25-hydroxyvitamin D and cancer mortality in the NHANES III study (1988-2006) | Insufficient data on studies |
| 159 | Edefonti 2010 | Nutrient-based dietary patterns and laryngeal cancer: Evidence from an exploratory factor analysis | Insufficient data on studies |
| 160 | Malodobra 2012 | VDR gene single nucleotide polymorphisms and their association with risk of oral cavity carcinoma | Insufficient data on studies |

**Supplementary Table 3.** General characteristics of 16 observational studies on vitamin D-related exposures and HNC.

| **Study** | **Study design** | **Location** | **Mean or median ±SD or range of follow-up, yrs** | **Male**  **(%)** | **Mean or median ±SD or range of age, yrs** | **Vitamin D exposure*** | **Vitamin D quantification method** | **Baseline Vitamin D** | **Outcomes** | **Cancer type and No. of cases** | **Cases** | **Controls/**  **Cohorts** | **Study**  **quality†** |
| --- | --- | --- | --- | --- | --- | --- | --- | --- | --- | --- | --- | --- | --- |
| Vitamin D receptor gene polymorphisms | | | | | | | | | | | | | |
| Zeljic  2012 | Hospital-based  case-control | Serbia | 2002-2007 | 74 | 58(36-81) | *VDR* polymorphisms  *FokI*, *TaqI*, *BsmI* | PCR–RFLP | NR | Oral cancer | Tongue(NR)  Foor of the mouth(NR) | 110 | 122 | 8 |
| Liu  2005 | Hospital-based  case-control | US | 1995-2003 | 76 | 57.1±11.9  Case  56.6±11.8  Control | *VDR* polymorphisms  *FokI*, *TaqI* | PCR–RFLP | NR | SCCHN | Oral cavity(NR)  Pharynx(NR)  Larynx(NR) | 719 | 821 | 9 |
| Huang  2011 | Hospital-based  case-control | China | 2005-2007 | 74 | 46.4±13.2  Case  44.4±13.4  Control | *VDR* polymorphisms  *FokI*, *BsmI* | PCR–RFLP | NR | NPC | Poorly differentiated SCC:144, Undifferentiated cancer:22,  Poorly differentiated adenocarcinoma:2,  Moderately differentiated SCC:6 | 171 | 176 | 9 |
| Bektas-Kayhan  2010 | Hospital-based  case-control | Turkey | 2006-2008 | 58 | 55.3±13.6  Case  57.2±12.8  Control | *VDR* polymorphism  *TaqI* | PCR–RFLP | NR | OSCC | Poor differentiated SCC:7  Moderate differentiated SCC:33  Well differentiated  SCC:20 | 64 | 87 | 7 |
| Vitamin D status | | | | | | | | | | | | | |
| Afzal  2013 | Population-based  cohort | Denmark | 21^a^(0.01-28) | 45 | 20-100 | Plasma  25(OH)D:  Per 50% decrease | CLIA(DiaSorin) | 41 ^a^ | HNC | Paranasal sinuses(NR) Salivary glands(NR)  Nasal cavity(NR)  Oral cavity(NR) Pharynx(NR)  Larynx(NR) | 122 | 9791 | 9 |
| Arem  2011 | Population-based  nested case-control | Finland | NR | 100 | 57(53-61) | Serum 25(OH)D:  Q1(<25.0)  Q4(50.0-<75.0) | CLIA(DiaSorin) | 31^a^ Case  32^a^ Control | HNC | Oral cavity:134  Pharynx:48  Larynx:158 | 340 | 340 | 9 |
| Bochen  2018 | Hospital-based  cohort | Germany | 1.75^a^ | 80 | 63 | Serum 25(OH)D:  <37.5  ≥37.5 | CLIA(DiaSorin) | 27.68^a^  Case  54.36^a^  Control | OS | Tonsil:80  Larynx:56  Hypopharynx:30  Base of tongue:26  Tongue border:22  Floor of the mouth:16  Cheek:1 | 89 | 231 | 7 |
| Weinstein  2018 | Population-based  cohort | Finland | 1985-2014 | 100 | 50-69 | Serum 25(OH)D:  Q1  Q5 | CLIA(DiaSorin) | 34.7^a^ | HNC mortality | NR | 126 | 398 | 9 |
| Skaaby  2014 | Population-based  cohort | Denmark | 11.3^a^ | 48 | 18-71 | Serum 25(OH)D:  Q1  Q4 | NICH ADV / HPLC (IDS) / Cobas E411 immunoassay | 52.4^b^ | HNC | NR | 44 | 12204 | 9 |
| Gugatschk  2011 | Hospital-based  cohort | Austria | 2006-2010 | 89 | 66 | Serum 25(OH)D:  NR | ELISA | 29.93^b^  Case  82.29^b^  Control | OS | Oral  cavity(NR)  Tongue(NR)  Tonsils(NR)  Mouth ﬂoor(NR) Larynx(NR) Hypopharynx(NR) | 29 | 88 | 8 |
| Fanidi  2016 | Population-based  nested case-control | European countries | 2004-2010 | 68 | 56.7(42-71) | Plasma 25(OH)D ：  50  25 | LC/MS | 42.4 ^a^  Case  46.1 ^a^  Control | HNC,  HNC mortality | Hypopharynx + Larynx:145  Gum + Oral cavity:110  Oropharynx:67  Head and neck other:28 | 350  145 | 940 | 9 |
| Meyer  2011 | Hospital-based  cohort | Canada | 8^b^ | 79 | 62.5±9.8 | Serum 25(OH)D:  Q1(<48)  Q4(>78) | RIA (DiaSorin) | 63.6^b^ | HNC mortality | Laryngeal cancer:450 | 223 | 522 | 9 |
| Giovannucci  2006 | Health professional-based  cohort | US | 1986-2000 | 100 | 40-75 | Plasma 25(OH)D ：  Per 25 increase | RIA | 22.8-90.8 | Oral/pharyngeal cancer | Oral/pharyngeal:51 | 51 | 47800 | 8 |
| Vitamin D intake | | | | | | | | | | | | | |
| Lipworth  2009 | Hospital-based  case-control | Italy | 1992-2005 | 68 | 58 | Dietary vitamin D:  Q1(<98)  Q3(>137) | FFQ | NR | HNC | Oral cavity:405  Pharynx:399 | 804 | 2080 | 7 |
| Negri  2000 | Hospital-based  case-control | Italy Switzerland | 1992-1997 | 75 | 57 | Dietary vitamin D:  FFQ  Q1  Q5 | FFQ | 120^b^ | Oral cavity and pharynx cancer | Oral cavity:344  Pharynx:410 | 754 | 1775 | 7 |
| Peters  2008 | Population-based case-control | US | 1999-2003 | 71 | NA | Total vitamin D:  Q1(<139)  Q5(>610) | self-administered 138 food item Harvard FFQ | 378.81^a^ | HNC | Oral:248  Larynx:97  Pharynx:151  Unknown:8 | 504 | 717 | 8 |
| Abbreviations: 25(OH)D, 25-hydroxyvitamin D; CLIA, chemiluminescence Immunoassay; DFS, disease-free survival; ELISA, enzyme linked immunosorbent assay; HNC, head and neck cancer; FFQ, Food Frequency Questionnaires; HPLC, high-performance liquid chromatography; LC, liquid chromatography; MS, mass spectrometry; NICH ADV, Nichols Advantage automated chemiluminescence assay; NPC, nasopharyngeal carcinoma; NA, not applicable; NR, not reported; OS, overall survival; OSCC, oral squamous cell carcinoma; RIA, radioimmunoassay.  ^a^Median  ^b^Mean  *Dietary vitamin D includes vitamin D from foods only and total vitamin D includes vitamin D from foods and supplements. Range of exposure indicates the cut points for the highest and lowest categories of daily vitamin D/ calcium intake. Median blood 25(OH)D levels in the lowest and highest categories or the cut points for the highest and lowest categories.  Vitamin D (IU/day), circulating blood 25-hydroxyvitamin D (nmol/L)  †Study quality was judged on the basis of the Newcastle-Ottawa Scale (1-9 stars). | | | | | | | | | | | | | |

**Supplementary Table 4.** Quality assessment of the included case-control studies by the Newcastle-Ottawa Scale (maximum score of 9).

|  | **Selection** | | | | **Comparability** | **Exposure** | | | **total** |
| --- | --- | --- | --- | --- | --- | --- | --- | --- | --- |
| **Study** | **1) Is the case definition adequate?** | **2) Represe ntativeness of the cases** | **3) Selection of Controls** | **4) Definition of Controls** | **1) Comparability of cases and controls on the basis of the design or analysis** | **1) Ascertainment of exposure** | **2) Same method of ascertainment for cases and controls** | **3) Non-Response rate** |  |
| Arem, 2011 | * | * | * | * | ** | * | * | * | 9 |
| Fanidi, 2016 | * | * | * | * | ** | * | * | * | 9 |
| Lipworth, 2009 | * | * | 0 | * | ** | 0 | * | * | 7 |
| Negri, 2000 | * | * | 0 | * | ** | 0 | * | * | 7 |
| Zeljic, 2012 | * | * | * | * | ** | * | 0 | * | 8 |
| Liu, 2005 | * | * | * | * | ** | * | * | * | 9 |
| Huang, 2011 | * | * | * | * | ** | * | * | * | 9 |
| Bektas-Kayhan, 2010 | 0 | * | 0 | * | ** | * | * | * | 7 |
| Peters, 2008 | * | * | * | * | ** | 0 | * | * | 8 |
| A star system is used to allow a semi-quantitative assessment of study quality, such that the highest quality studies are awarded a maximum of one star for each item with the exception of the item related to comparability that allows the assignment of two stars. The NOS ranges between zero up to nine stars. | | | | | | | | | |

**Supplementary Table 5.** Quality assessment of the included cohort studies by the Newcastle-Ottawa Scale (maximum score of 9).

|  | **Selection** | | | | **Comparability** | **Exposure** | | | **total** |
| --- | --- | --- | --- | --- | --- | --- | --- | --- | --- |
| **Study** | **1) Representativeness of the exposed cohort** | **2) Selection of the non exposed cohort** | **3) Ascertainment of exposure** | **4) Demonstration that outcome of interest was not present at start of study** | **1) Comparability of cohorts on the basis of the design or analysis** | **1) Assessment of outcome** | **2) Was follow-up long enough for outcomes to occur** | **3) Adequacy of follow up of cohorts** |  |
| Afzal, 2013 | * | * | * | * | ** | * | * | * | 9 |
| Bochen, 2018 | * | * | * | * | 0 | * | * | * | 7 |
| Weinstein, 2018 | * | * | * | * | ** | * | * | * | 9 |
| Skaaby, 2014 | * | * | * | * | ** | * | * | * | 9 |
| Gugatschka, 2011 | * | * | * | * | * | * | * | * | 8 |
| Meyer, 2011 | * | * | * | * | ** | * | * | * | 9 |
| Giovannucci, 2006 | 0 | * | * | * | ** | * | * | * | 8 |
| A star system is used to allow a semi-quantitative assessment of study quality, such that the highest quality studies are awarded a maximum of one star for each item with the exception of the item related to comparability that allows the assignment of two stars. The NOS ranges between zero up to nine stars. | | | | | | | | | |

**Supplementary Table 6.** List of adjustment factors employed in the 16 observational studies.

| **Study** | **Location** | **Method of adjustment** | **Adjustment factors** |
| --- | --- | --- | --- |
| Vitamin D receptor gene polymorphisms | | | |
| Zeljic, 2012 | Serbia | Unconditional logistic regression model | Age, sex, smoking status, alcohol consumption |
| Liu, 2005 | US | Logistic regression model | Age, sex, smoking status, alcohol use |
| Huang, 2011 | China | Adjusted method not specified | Age, sex |
| Bektas-Kayhan, 2010 | Turkey | Adjusted method not specified | Age, sex, smoking status, family history of cancer, nodal metastasis, differentiation, grade of the tumor |
| Vitamin D status | | | |
| Afzal, 2013 | Denmark | Cox proportional hazards regression model | Age, smoking, alcohol intake, BMI, education level, physical activity, leisure time |
| Arem, 2011 | Finland | Multiple logistic regression model | Age, smoking, alcohol intake, pack-years, BMI, physical activity, history of hypertension, history of diabetes, dentures, missing teeth, daily calorie intake, fish intake, serum retinol, serum alpha tocopherol, serum beta carotene, serum cholesterol, HDL, season of blood draw, marital status, vacations to sunny areas |
| Bochen, 2018 | Germany | Kaplan-Meier model | NR |
| Weinstein, 2018 | Finland | Cox proportional hazards regression model | Age, smoking, BMI, physical activity, serum cholesterol, history of diabetes, family history of cancer, systolic blood pressure, trial intervention group, calendar year of diagnosis |
| Skaaby, 2014 | Denmark | Cox proportional hazards regression model | Sex, BMI, education, season during which blood was drawn, study, physical activity, smoking habits, alcohol intake, intake of ﬁsh |
| Gugatschka, 2011 | Austria | Multivariate Cox-regression model | BMI, tumor size, renal disease |
| Fanidi, 2016 | European countries | Unconditional logistic regression / Cox proportional hazards model | Age, country, sex, smoking, BMI, seasonality, educational attainment, cotinine quartiles, alcohol intake |
| Meyer, 2011 | Canada | Multivariate Cox proportional hazard model | Age, smoking, alcohol consumption, BMI, site, stage, season of blood collection |
| Giovannucci, 2006 | US | Multivariate Cox proportional hazard model | Age, height, smoking, alcohol, intakes of total calories, red meat, calcium, retinol, total fruits and vegetables |
| Vitamin D intake | | | |
| Lipworth, 2009 | Italy | Unconditional multiple logistic regression model | Age, smoking, alcohol consumption, sex, study center, education, nonalcohol energy intake |
| Negri, 2000 | Italy Switzerland | Unconditional multiple logistic regression model | Age, sex, smoking, drinking, center, education, occupation, BMI, non‐alcohol energy intake |
| Peters, 2008 | US | Unconditional logistic regression model | Age, race, sex |
| Abbreviations: BMI, body mass index; HDL, high-density lipoprotein. | | | |

**Supplementary Table 7.** Subgroup analysis of the effect of vitamin D status and intake on HNC incidence.

| **Subgroup title** | **No of studies** | **No of participants** | ***I^2^* (%)** | **Odds ratio (95% CI)** | ***P* for interaction** |
| --- | --- | --- | --- | --- | --- |
| **Vitamin D status** | | | | | |
| Overall | 5 | 71 765 | 0 | 0.68 (0.59 to 0.78) | — |
| Geographic region of study | | | | | |
| European | 4 | 23 965 | 0 | 0.69 (0.60 to 0.79) | 0.11 |
| North America | 1 | 47 800 | — | 0.30 (0.11 to 0.81) |  |
| Sex | | | | | |
| Male | 2 | 48 480 | 29 | 0.44 (0.14 to 1.36) | 0.44 |
| Male and female | 3 | 23 285 | 0 | 0.69 (0.60 to 0.79) |  |
| Sample | | | | | |
| Serum | 2 | 12 884 | 0 | 0.55 (0.23 to 1.36) | 0.67 |
| Plasma | 3 | 58 881 | 25 | 0.68 (0.56 to 0.81) |  |
| Assay method | | | | | |
| Radioimmunoassay | 1 | 47 800 | — | 0.30 (0.11 to 0.81) | 0.26 |
| Automated assay | 2 | 10 471 | 0 | 0.69 (0.58 to 0.83) |  |
| Chromatographic | 1 | 1290 | — | 0.70 (0.56 to 0.88) |  |
| No of participants | | | | | |
| ≥2000 | 3 | 69 795 | 36 | 0.56 (0.35 to 0.90) | 0.38 |
| ＜2000 | 2 | 1970 | 0 | 0.70 (0.56 to 0.88) |  |
| No of events | | | | | |
| ≥200 | 2 | 1970 | 0 | 0.70 (0.56 to 0.88) | 0.38 |
| ＜200 | 3 | 69 795 | 36 | 0.56 (0.35 to 0.90) |  |
| Population source | | | | | |
| Community | 4 | 23 965 | 0 | 0.69 (0.60 to 0.79) | 0.11 |
| Health professional | 1 | 47 800 | — | 0.30 (0.11 to 0.81) |  |
| Controlled for seasonality | | | | | |
| Yes | 3 | 14 174 | 0 | 0.69 (0.55 to 0.86) | 0.51 |
| No | 2 | 57 591 | 61 | 0.53 (0.25 to 1.13) |  |
| Risk of bias score† | | | | | |
| 9 | 4 | 23 965 | 0 | 0.69 (0.60 to 0.79) | 0.11 |
| ＜9 | 1 | 47 800 | — | 0.30 (0.11 to 0.81) |  |
| Year of publication | | | | | |
| Before 2015 | 4 | 70 475 | 11 | 0.62 (0.46 to 0.85) | 0.56 |
| In or after 2015 | 1 | 1290 | — | 0.70 (0.56 to 0.88) |  |
| Study design | | | | | |
| Cohort studies | 3 | 69 795 | 36 | 0.56 (0.35 to 0.90) | 0.38 |
| Case-control studies | 2 | 1970 | 0 | 0.70 (0.56 to 0.88) |  |
| **Vitamin D intake** | | | | | |
| Overall | 3 | 6634 | 0 | 0.77 (0.65 to 0.92) | — |
| Geographic region of study | | | | | |
| European | 2 | 5413 | 0 | 0.74 (0.61 to 0.90) | 0.43 |
| North America | 1 | 1221 | — | 0.88 (0.60 to 1.28) |  |
| Sex‡ | | | | | |
| Male | 2 | 3851 | 0 | 0.78 (0.65 to 0.94) | 0.23 |
| Female | 2 | 1562 | 8 | 1.00 (0.71 to 1.40) |  |
| Sample | | | | | |
| Dietary | 2 | 5413 | 0 | 0.74 (0.61 to 0.90) | 0.43 |
| Dietary and supplements | 1 | 1221 | — | 0.88 (0.60 to 1.28) |  |
| Follow-up (years) | | | | | |
| ≥5 | 2 | 5413 | 0 | 0.74 (0.61 to 0.90) | 0.43 |
| ＜5 | 1 | 1221 | — | 0.88 (0.60 to 1.28) |  |
| No of participants | | | | | |
| ≥2000 | 2 | 5413 | 0 | 0.74 (0.61 to 0.90) | 0.43 |
| ＜2000 | 1 | 1221 | — | 0.88 (0.60 to 1.28) |  |
| No of events | | | | | |
| ≥700 | 2 | 5413 | 0 | 0.74 (0.61 to 0.90) | 0.43 |
| ＜700 | 1 | 1221 | — | 0.88 (0.60 to 1.28) |  |
| Population source | | | | | |
| Community | 1 | 1221 | — | 0.88 (0.60 to 1.28) | 0.43 |
| Hospital | 2 | 5413 | 0 | 0.74 (0.61 to 0.90) |  |
| †Based on Newcastle-Ottawa scale. ‡Based on available studies with relevant subgroup information. | | | | | |

**Supplementary Table 8.** Summary ORs and 95% CI of *FokI*, *TaqI*, and *BsmI* polymorphisms and head and neck cancer risk.

| ***FokI*** | **N** | ***f* vs. *F*** | | | ***ff* vs. *FF*** | | | ***Ff* vs. *FF*** | | | ***Ff*+*ff* vs. *FF*** | | | | ***ff* vs. *Ff*+*FF*** | | |
| --- | --- | --- | --- | --- | --- | --- | --- | --- | --- | --- | --- | --- | --- | --- | --- | --- | --- |
|  |  | **OR (95%CI)** | ***P*** | ***I^2^*** | **OR (95%CI)** | ***P*** | ***I^2^*** | **OR (95%CI)** | ***P*** | ***I^2^*** | **OR (95%CI)** | | ***P*** | ***I^2^*** | **OR (95%CI)** | ***P*** | ***I^2^*** |
| Total | 3 | 0.94(0.76-1.18) | 0.61 | 53 | 0.75(0.58-0.97) | 0.03 | 31 | 0.95(0.79-1.15) | 0.62 | 47 | 1.02(0.71-1.45) | | 0.92 | 59 | 0.77(0.61-0.97) | 0.03 | 0 |
| Ethnicity | | | | | | | | | | | | | | | | | |
| Caucasian | 2 | 0.93(0.67-1.30) | 0.68 | 66 | 0.72(0.48-1.09) | 0.12 | 20 | 1.09(0.62-1.91) | 0.76 | 72 | 1.03(0.58-1.84) | | 0.92 | 75 | 0.72(0.55-0.94) | 0.02 | 0 |
| Asian | 1 | 1.03(0.77-1.39) | 0.84 | — | 1.05(0.59-1.86) | 0.87 | — | 1.07(0.65-1.76) | 0.80 | — | 1.10(0.70-1.74) | | 0.68 | — | 0.98(0.60-1.59) | 0.92 | — |
| Follow-up (years) | | | | | | | | | | | | | | | | | |
| ≥5 | 2 | 0.93(0.67-1.30) | 0.68 | 66 | 0.72(0.48-1.09) | 0.12 | 20 | 1.09(0.62-1.91) | 0.76 | 72 | 1.03(0.58-1.84) | | 0.92 | 75 | 0.72(0.55-0.94) | 0.02 | 0 |
| ＜5 | 1 | 1.03(0.77-1.39) | 0.84 | — | 1.05(0.59-1.86) | 0.87 | — | 1.07(0.65-1.76) | 0.80 | — | 1.10(0.70-1.74) | | 0.68 | — | 0.98(0.60-1.59) | 0.92 | — |
| Age (years) | | | | | | | | | | | | | | | | | |
| ≥50 | 2 | 0.93(0.67-1.30) | 0.68 | 66 | 0.72(0.48-1.09) | 0.12 | 20 | 1.09(0.62-1.91) | 0.76 | 72 | 1.03(0.58-1.84) | | 0.92 | 75 | 0.72(0.55-0.94) | 0.02 | 0 |
| ＜50 | 1 | 1.03(0.77-1.39) | 0.84 | — | 1.05(0.59-1.86) | 0.87 | — | 1.07(0.65-1.76) | 0.80 | — | 1.10(0.70-1.74) | | 0.68 | — | 0.98(0.60-1.59) | 0.92 | — |
| Risk of bias score | | | | | | | | | | | | | | | | | |
| 9 | 2 | 0.88(0.71-1.10) | 0.27 | 48 | 0.78(0.50-1.21) | 0.26 | 51 | 0.90(0.73-1.09) | 0.28 | 0 | 0.88(0.67-1.15) | | 0.35 | 32 | 0.78(0.58-1.04) | 0.09 | 20 |
| ＜9 | 1 | 1.03(0.77-1.39) | 0.84 | — | 1.13(0.45-2.80) | 0.79 | — | 1.55(0.88-2.74) | 0.13 | — | 1.48(0.85-2.56) | | 0.17 | — | 0.86(0.37-1.98) | 0.72 | — |
| Cancer site† | | | | | | | | | | | | | | | | | |
| Oral cavity | 2 | — | — | — | 0.69(0.32-1.48) | 0.34 | 55 | 1.14(0.70-1.85) | 0.61 | 57 | — | — | — | — | — | — | — |
| Pharynx | 2 | — | — | — | 0.79(0.50-1.25) | 0.31 | 45 | 0.86(0.67-1.10) | 0.23 | 0 | — | — | — | — | — | — | — |
| *TaqI* |  | *t* vs. *T* | | | *tt* vs. *TT* | | | *Tt* vs. *TT* | | | *Tt*+*tt* vs. *TT* | | | | *tt* vs. *Tt*+*TT* | | |
| Total | 3 | 0.90(0.79-1.03) | 0.12 | 24 | 0.72(0.55-0.95) | 0.02 | 0 | 1.30(0.83-2.04) | 0.25 | 62 | 1.14(0.77-1.69) | | 0.52 | 57 | 0.70(0.55-0.90) | 0.01 | 0 |
| Ethnicity | | | | | | | | | | | | | | | | | |
| Caucasian | 2 | 0.98(0.71-1.35) | 0.89 | 62 | 0.74(0.56-0.98) | 0.04 | 0 | 1.22(0.69-2.16) | 0.49 | 74 | 1.12(0.65-1.95) | | 0.68 | 74 | 0.73(0.56-0.95) | 0.02 | 0 |
| Asian | 1 | 0.90(0.56-1.42) | 0.64 | — | 0.54(0.18-1.61) | 0.27 | — | 1.67(0.81-3.46) | 0.16 | — | 1.31(0.66-2.62) | | 0.44 | — | 0.40(0.15-1.06) | 0.07 | — |
| Follow-up (years) | | | | | | | | | | | | | | | | | |
| ≥5 | 2 | 0.98(0.71-1.35) | 0.89 | 62 | 0.74(0.56-0.98) | 0.04 | 0 | 1.22(0.69-2.16) | 0.49 | 74 | 1.12(0.65-1.95) | | 0.68 | 74 | 0.73(0.56-0.95) | 0.02 | 0 |
| ＜5 | 1 | 0.90(0.56-1.42) | 0.64 | — | 0.54(0.18-1.61) | 0.27 | — | 1.67(0.81-3.46) | 0.16 | — | 1.31(0.66-2.62) | | 0.44 | — | 0.40(0.15-1.06) | 0.07 | — |
| Source of controls | | | | | | | | | | | | | | | | | |
| Population | 2 | 0.98(0.71-1.35) | 0.89 | 62 | 0.74(0.56-0.98) | 0.04 | 0 | 1.22(0.69-2.16) | 0.49 | 74 | 1.12(0.65-1.95) | | 0.68 | 74 | 0.73(0.56-0.95) | 0.02 | 0 |
| Hospital | 1 | 0.90(0.56-1.42) | 0.64 | — | 0.54(0.18-1.61) | 0.27 | — | 1.67(0.81-3.46) | 0.16 | — | 1.31(0.66-2.62) | | 0.44 | — | 0.40(0.15-1.06) | 0.07 | — |
| Risk of bias score | | | | | | | | | | | | | | | | | |
| 9 | 1 | 0.86(0.75-1.00) | 0.05 | — | 0.71(0.52-0.96) | 0.03 | — | 0.96(0.77-1.20) | 0.74 | — | 0.89(0.72-1.10) | | 0.28 | — | 0.72(0.55-0.95) | 0.02 | — |
| ＜9 | 2 | 1.07(0.80-1.44) | 0.64 | 0 | 0.81(0.41-1.61) | 0.55 | 0 | 1.71(1.11-2.66) | 0.02 | 0 | 1.47(0.97-2.24) | | 0.07 | 0 | 0.59(0.30-1.16) | 0.13 | 10 |
| Cancer site | | | | | | | | | | | | | | | | | |
| Oral | 2 | 1.07(0.80-1.44) | 0.64 | 0 | 0.81(0.41-1.61) | 0.55 | 0 | 1.71(1.11-2.66) | 0.02 | 0 | 1.47(0.97-2.24) | | 0.07 | 0 | 0.59(0.30-1.16) | 0.13 | 10 |
| HNC | 1 | 0.86(0.75-1.00) | 0.05 | — | 0.71(0.52-0.96) | 0.03 | — | 0.96(0.77-1.20) | 0.74 | — | 0.89(0.72-1.10) | | 0.28 | — | 0.72(0.55-0.95) | 0.02 | — |
| *BsmI* |  | *B* vs. *b* | | | *BB* vs. *bb* | | | *Bb* vs. *BB* | | | *Bb*+*bb* vs. *BB* | | | | *bb* vs. *Bb*+*bb* | | |
| Total | 2 | 1.03(0.64-1.67) | 0.90 | 55 | 0.28(0.05-1.68) | 0.16 | 0 | 1.25(0.61-2.57) | 0.54 | 70 | 1.18(0.57-2.45) | | 0.65 | 72 | 0.24(0.04-1.44) | 0.12 | 0 |
| †Based on available studies with relevant subgroup information. | | | | | | | | | | | | | | | | | |

**Supplementary Table 9.** Subgroup analysis of the effect of vitamin D status on HNC morality.

| **Subgroup title** | **No of studies** | **No of participants** | ***I^2^* (%)** | **Odds ratio (95% CI)** | ***P* for interaction** |
| --- | --- | --- | --- | --- | --- |
| Overall | 3 | 2210 | 0 | 0.75 (0.60 to 0.94) | — |
| Geographic region of study | | | | | |
| European | 2 | 1688 | 0 | 0.72 (0.55 to 0.93) | 0.49 |
| North America | 1 | 522 | — | 0.85 (0.57 to 1.27) |  |
| Sex | | | | | |
| Male | 1 | 398 | — | 0.74 (0.42 to 1.30) | 0.94 |
| Male and female | 2 | 1812 | 0 | 0.76 (0.60 to 0.96) |  |
| Sample | | | | | |
| Serum | 2 | 920 | 0 | 0.81 (0.58 to 1.13) | 0.56 |
| Plasma | 1 | 1290 | — | 0.71 (0.53 to 0.96) |  |
| No of participants | | | | | |
| ≥500 | 2 | 1812 | 0 | 0.76 (0.60 to 0.96) | 0.94 |
| ＜500 | 1 | 398 | — | 0.74 (0.42 to 1.30) |  |
| No of events | | | | | |
| ≥200 | 1 | 522 | — | 0.85 (0.57 to 1.27) | 0.49 |
| ＜200 | 2 | 1688 | 0 | 0.72 (0.55 to 0.93) |  |
| Population source | | | | | |
| Community | 2 | 1688 | 0 | 0.72 (0.55 to 0.93) | 0.49 |
| Hospital | 1 | 522 | — | 0.85 (0.57 to 1.27) |  |
| Controlled for seasonality | | | | | |
| Yes | 2 | 1812 | 0 | 0.76 (0.60 to 0.96) | 0.94 |
| No | 1 | 398 | — | 0.74 (0.42 to 1.30) |  |
| Year of publication | | | | | |
| Before 2015 | 1 | 522 | — | 0.85 (0.57 to 1.27) | 0.49 |
| In or after 2015 | 2 | 1688 | 0 | 0.72 (0.55 to 0.93) |  |
| Study design | | | | | |
| Cohort studies | 2 | 920 | 0 | 0.81 (0.58 to 1.13) | 0.56 |
| Case-control studies | 1 | 1290 | — | 0.71 (0.53 to 0.96) |  |

## Supplementary Figures

**
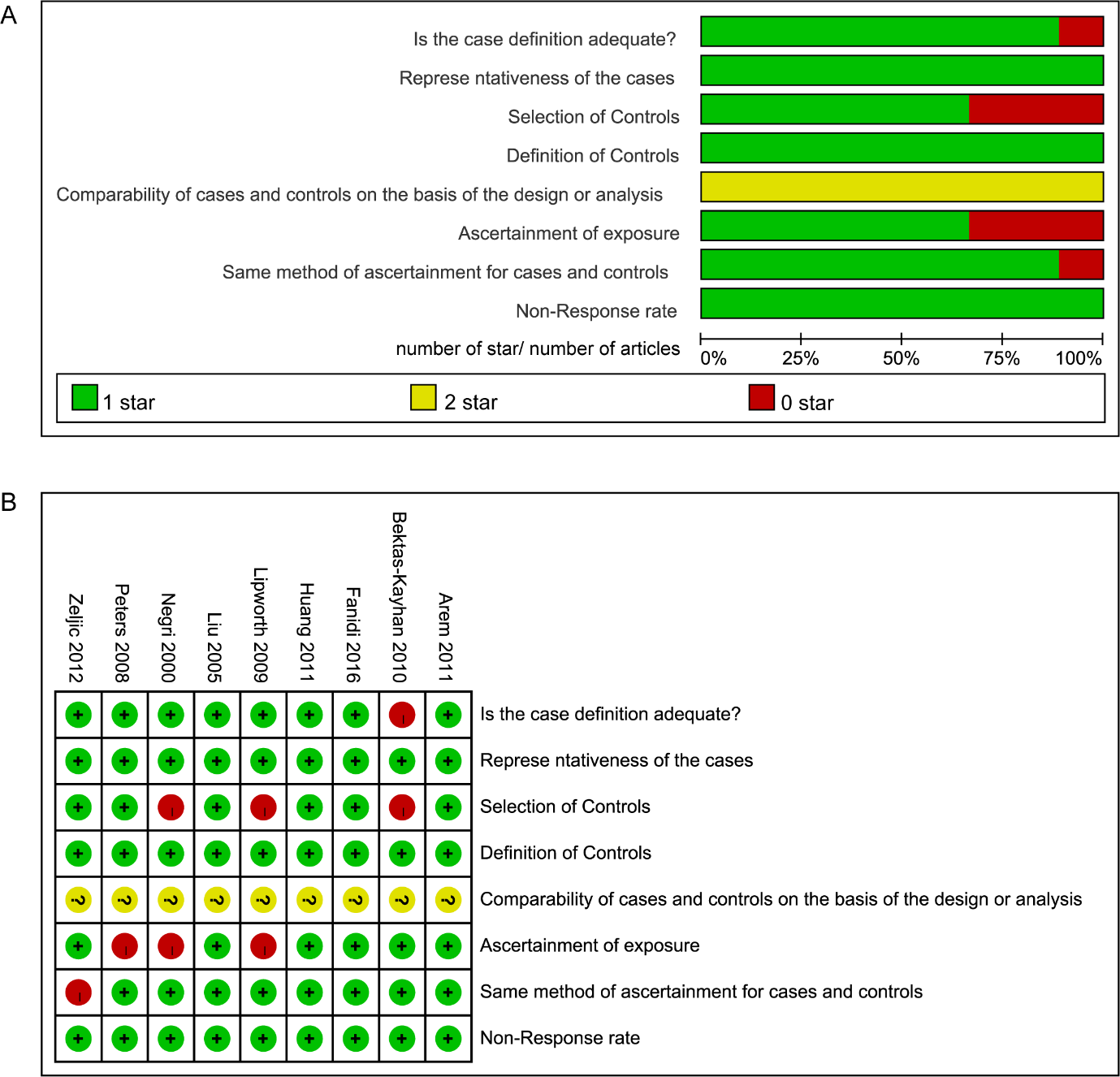
**

**Supplementary Figure 1.** Risk of bias graph and summary in case-control studies.

**
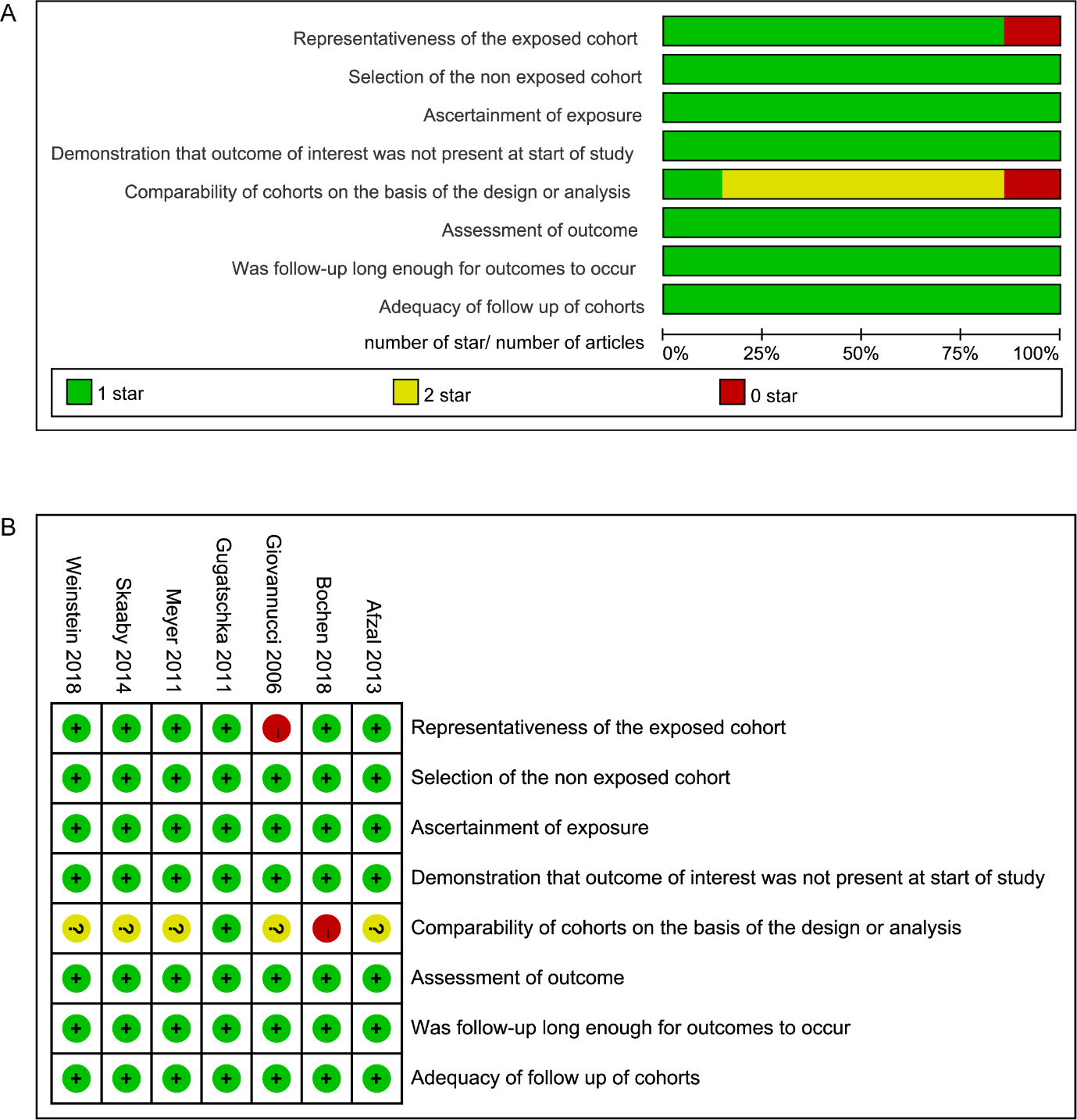
**

**Supplementary Figure 2.** Risk of bias graph and summary in cohort studies.


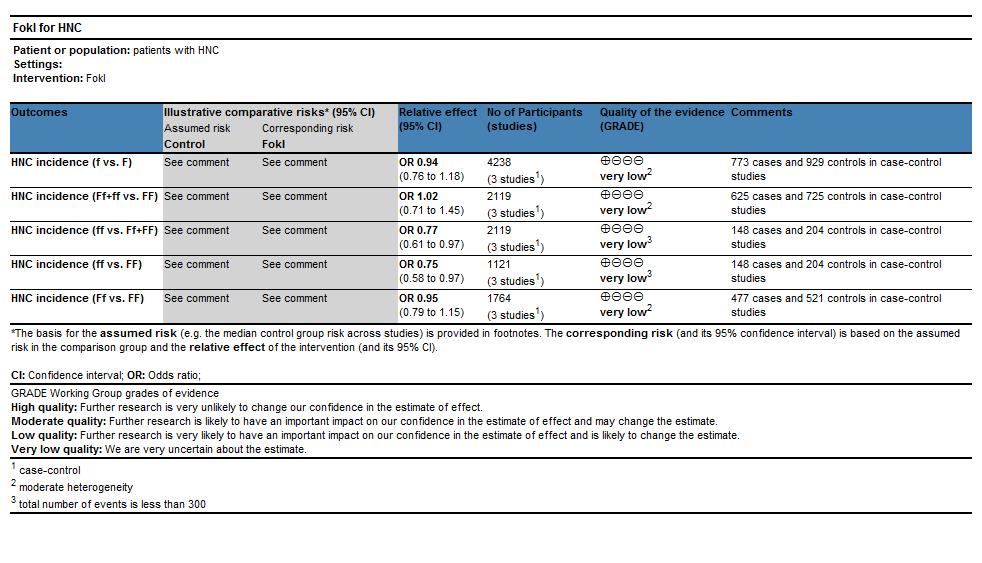


**Supplementary Figure 3.** GRADE summary of findings for the association of *FokI* polymorphism and head and neck cancer risk.


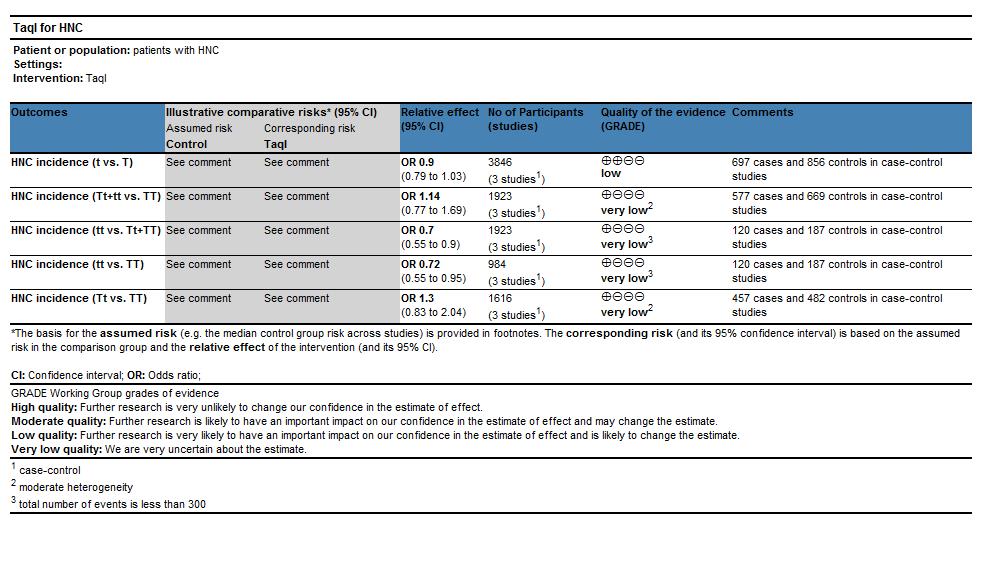


**Supplementary Figure 4.** GRADE summary of findings for the association of *TaqI* polymorphism and head and neck cancer risk.


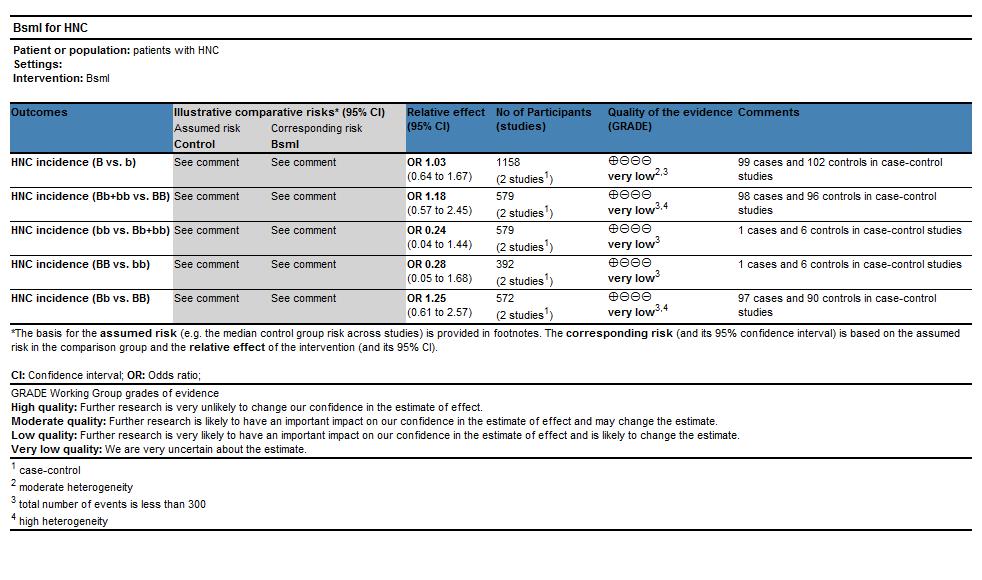


**Supplementary Figure 5.** GRADE summary of findings for the association of *BsmI* polymorphism and head and neck cancer risk.


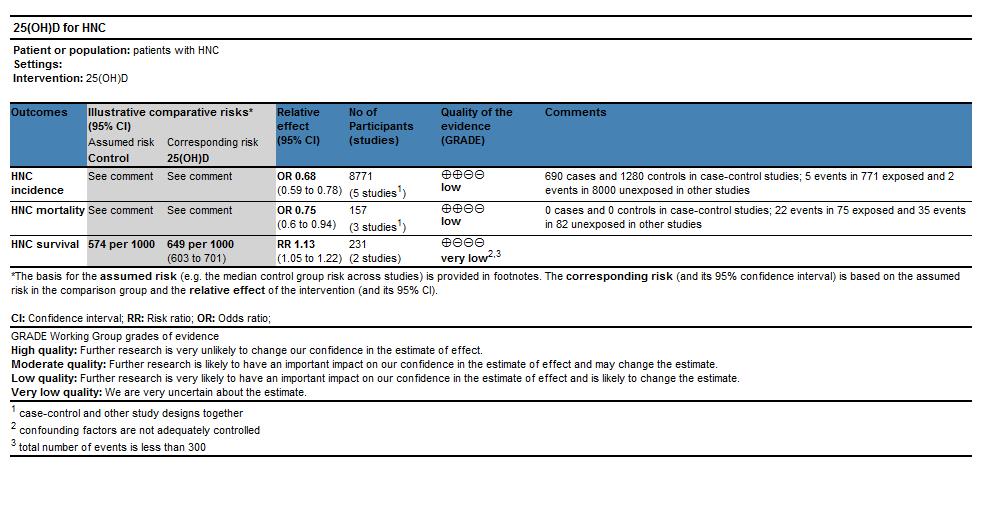


**Supplementary Figure 6.** GRADE summary of findings for the associations of vitamin D status on the incidence, morality, and survival of HNC.


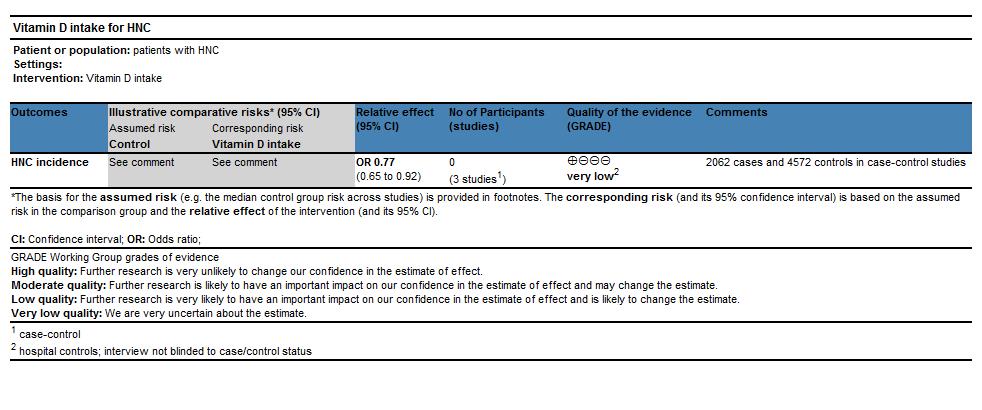


**Supplementary Figure 7.** GRADE summary of findings for the associations of vitamin D intake on the incidence of HNC.
